# Fully integrated parity–time-symmetric electronics

**DOI:** 10.1038/s41565-021-01038-4

**Published:** 2022-03-17

**Authors:** Weidong Cao, Changqing Wang, Weijian Chen, Song Hu, Hua Wang, Lan Yang, Xuan Zhang

**Affiliations:** 1 Department of Electrical and Systems Engineering, Washington University, St. Louis, MO USA; 2 Department of Physics, Washington University, St. Louis, MO USA; 3 Center for Quantum Sensors, Washington University, St. Louis, MO USA; 4 School of Electrical and Computer Engineering, Georgia Institute of Technology, Atlanta, GA USA; 5 Department of Information Technology and Electrical Engineering, Swiss Federal Institute of Technology, Zurich, Switzerland; 6 Present Address: Apple, Cupertino, CA USA

**Keywords:** Electrical and electronic engineering, Design, synthesis and processing

## Abstract

Harnessing parity–time symmetry with balanced gain and loss profiles has created a variety of opportunities in electronics from wireless energy transfer to telemetry sensing and topological defect engineering. However, existing implementations often employ ad hoc approaches at low operating frequencies and are unable to accommodate large-scale integration. Here we report a fully integrated realization of parity–time symmetry in a standard complementary metal–oxide–semiconductor process technology. Our work demonstrates salient parity–time symmetry features such as phase transition as well as the ability to manipulate broadband microwave generation and propagation beyond the limitations encountered by existing schemes. The system shows 2.1 times the bandwidth and 30% noise reduction compared to conventional microwave generation in the oscillatory mode, and displays large non-reciprocal microwave transport from 2.75 to 3.10 GHz in the non-oscillatory mode due to enhanced nonlinearities. This approach could enrich integrated circuit design methodology beyond well-established performance limits and enable the use of scalable integrated circuit technology to study topological effects in high-dimensional non-Hermitian systems.

## Abstract (web summary)

The implementation of parity–time symmetry in a complementary metal–oxide–semiconductor process technology enables the realization of wide-band high-quality microwave generation and broadband strong microwave isolation at gigahertz frequencies.

## Main

Symmetry is one of the most essential notions to influence the fundamental properties of physical systems. Quantum systems whose Hamiltonians commute with a joint parity–time (PT) operator (${\mathrm{PT}}\hat{H}=\hat{H}{\mathrm{PT}}$) possess a special kind of symmetry, known as PT symmetry. In general, open quantum systems interacting with environments can be described by non-Hermitian Hamiltonians, which preserve complex eigenvalues. However, a system with PT symmetry possesses purely real eigenspectra in certain regimes, whereas the eigenstates are non-orthogonal to each other. Over the past few years, PT-symmetric systems featuring balanced gain and loss profiles have been studied in optics^1–15^, optomechanics^16,17^, optoelectronics^18^ and acoustics^19–21^ and have initiated a number of exotic effects and applications including electromagnetically induced transparency^1,2^, coherent perfect absorption-lasing^3–6^, topological light steering^7^, single-mode lasing^8–10^, ultrasensitive sensors^11–13^, opto-electronic microwave generation^18^ and non-reciprocal photon^14,15^ and phonon^21^ transmission.

Electronics has recently emerged as a promising field to study PT symmetry due to the flexibility and reliability of controlling active and passive electronic resonators^22–31^. Experiments have been conducted on printed circuit boards^22–30^ and in microelectromechanical systems^31^, showing robust wireless energy transfer^25,26^, enhanced telemetry sensing^27,31^ and topological effects^28–30^. However, these electronic platforms are confined to low-frequency operation below a few hundred megahertz and are difficult to scale to small physical dimensions and complex integrated structures. To explore and unleash the full potential of PT symmetry in electronics, one must look beyond existing ad hoc implementation approaches. Integrated circuit (IC) technology—the leading nanotechnology for electronics—provides a standard manufacturing process for flexible and customized designs that consist of millions of nanoscale integrated devices. Its scalability in physical dimension enables ICs to be a powerful platform that covers a wide applied spectra from d.c. to terahertz. It also supports integration of complex three-dimensional structures^32^, allowing one to extend a core electronic non-Hermitian unit into higher-dimensional structures to study topological electronics^28–30^. Despite such intriguing properties, IC technology has yet to be employed to realize PT symmetry, although gain, loss and their coupling effects do commonly exist in ICs.

On the other hand, as has been shown in the field of optics and acoustics, PT symmetry can provide enhanced ability in wave generation^18^ and propagation^14,15,21^, making it especially attractive for IC technology. Effective implementations of these functionalities in the microwave domain remain challenging in ICs, and the capability to exceed the conventional performance limits has long been sought after. In particular, electrical non-reciprocal microwave transmission is highly desirable for diverse on-chip applications^33^, yet the existing approach that uses bulky and costly ferromagnetic devices suffers from a number of drawbacks and is incompatible with the semiconductor fabrication process^34^. Advancing integrated magnetic-free non-reciprocal devices thus not only demands a breakthrough in materials and fabrication technologies, but also relies on our ability to enrich the arsenal of IC design methodology. PT-symmetric systems can break Lorentz reciprocity to produce enhanced non-reciprocity in the presence of nonlinearity^35^. Such a merit has been demonstrated in acoustic wave^21^ and light transmissions^14,15^, but remains unexplored in electronics. Therefore, harnessing PT symmetry for broadband microwave generation and non-reciprocity with chip-scale implementation is particularly appealing and has immense potential.

In this Article, we report a fully integrated implementation of PT symmetry in a 130 nm complementary metal–oxide–semiconductor (CMOS) process technology, and use it to create wide-band high-quality microwave generation and broadband strong microwave isolation at the gigahertz scale. We demonstrate the PT symmetry phase transition feature on this scalable platform. With the distinctive gain–loss tuning freedom, we show that in oscillatory mode, our system exhibits a wide-band microwave generation from 2.63 GHz to 3.20 GHz with an average −120 dBc Hz^–1^ noise intensity (where dBc represents the decibels relative to the carrier), achieving 2.1 times the bandwidth with 70% of the phase noise compared to a baseline conventional oscillator. While in non-oscillatory mode, the intrinsic nonlinearity of the system is greatly enhanced by the PT symmetry, leading to 7–21 dB non-reciprocal microwave transmission in a broad band of 2.75–3.10 GHz. Our results show that the introduction of PT symmetry into IC technology can benefit a broad range of chip-based applications including waveform synthesis and generation^36^, frequency modulation^37^ and the manipulation of microwave propagation^38^.

## Fully integrated PT-symmetric electronic system

**Fig. 1:**
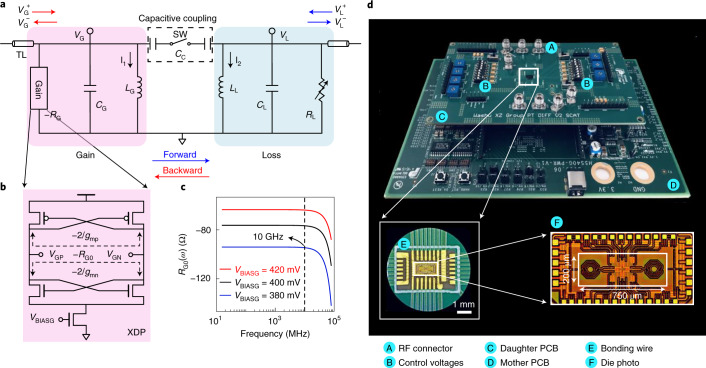
Illustration and characterization of a fully integrated PT-symmetric electronic system. **a**, The schematic diagram of the fully integrated PT-symmetric electronic system, where capacitive coupling (*C*_C_) is used to connect two RLC resonators, one with gain −*R*_G_ and the other one with equal loss (*R*_L_). The two units can be coupled and decoupled by the switch SW. The forward transmission is defined from gain side to loss side. **b**, A XDP is used in our design to generate −*R*_G0_, which is defined as the reciprocal of the total small-signal transconductance of the PMOS differential pair and NMOS differential pair; that is, –*R*_G0_ = –2/(*g*_mp_ + *g*_mn_). **c**, The simulation results show that the negative resistance −*R*_G0_ remains constant in a wide frequency range up to 10 GHz. **d**, Fully integrated PT-symmetric electronic system. The chip is wire-bonded on a daughter printed circuit board (PCB), which provides various control voltages and RF connectors for testing. A mother PCB supplies powers to the daughter PCB. The fully integrated PT-symmetric electronic system is fabricated in a 130 nm CMOS process technology whose core area is 200 × 750 μm^2^. PMOS, p-type metal–oxide–semiconductor; NMOS: n-type metal–oxide–semiconductor; V_GP_ and V_GN_ are the differential outputs of the XDP; V_BIASG_ is the control voltage of the XDP; *g*_mp_ and *g*_mn_ are the small-signal transconductances generated by the PMOS differential pair and NMOS differential pair, respectively.

Our fully integrated PT-symmetric electronic system consists of two capacitively coupled resistor–inductor–capacitor (RLC) resonators, one with gain and the other one with equivalent loss (Fig. 1a). A differential architecture (Supplementary Fig. 1) is implemented to obtain superior device matching, better robustness and higher reliability against noise, interference and failure. The equivalent single-ended circuit schematic, shown in Fig. 1a, is used to simplify analysis. We propose a cross-coupled differential pair^39^ (XDP) as a negative resistance converter (NRC) in our system (Fig. 1b), which generates a stable and tunable gain beyond 10 GHz (Fig. 1c). It can also better conserve energy than operational-amplifier-based NRCs^21–27^ and Colpitts-type-based NRCs^31^, while generating the same amount of gain. These characteristics are ideal for broadband and energy-efficient microwave PT-symmetric electronic systems.

The active RLC resonator has a gain rate −*R*_G0_ generated by the XDP, a variable loss rate *R*_G1_ and an intrinsic loss rate *R*_G2_, yielding a total gain of −*R*_G_ = −*R*_G0_ ∣∣ *R*_G1_ ∣∣ *R*_G2_. The total loss rate *R*_L_ = *R*_L0_ ∣∣ *R*_L1_ in the passive RLC resonator is contributed by a variable loss *R*_L0_ and an intrinsic loss *R*_L1_. The capacitance in each RLC resonator comes from a fixed metal–insulator–metal (MIM) capacitor and a varactor. The coupling capacitor consists of two equal MIM capacitors in serial connection via an on-chip switch (Fig. 1a). The inductance in each RLC resonator is provided by a symmetrical parallel inductor (Supplementary Fig. 2). Both RLC resonators have the same natural frequency *ω*_0_ = 3.20 GHz. The system is integrated on one monolithic chip with a core area of 200 × 750 μm^2^ using a standard 130 nm CMOS process technology (Fig. 1d). Note that alternative fully integrated PT-symmetric structures could also be implemented (Supplementary Section 7). To show the functionalities and performances, we bonded the chip on a test board with gold wires (Fig. 1d). The board provides a power supply, control voltages and radio-frequency (RF) connectors for measurements. The gain and loss rate and the varactor capacitance can be flexibly adjusted by the control voltages. The PT symmetry condition is satisfied by setting *R*_G_ ≈ *R*_L_ = *R*, *L*_G_ ≈ *L*_L_ = *L* and *C*_G_ ≈ *C*_L_ = *C*, where *R* is the resistance value, *L*_G_ (*L*_L_) is the inductance value on the gain (loss) side and *C*_G_ (*C*_L_) is the capacitance value on the gain (loss) side. The detailed circuit parameters are provided in the Methods.

## PT symmetry phase transition

We first numerically analyse the PT symmetry transition of our system based on the small-signal model—a common methodology in analysing analogue circuits. The system is considered as a linear time-invariant system, which is valid for small-signal inputs. By applying Kirchoff’s law on the equivalent circuit (Fig. 1a), four eigenfrequencies^22^ are found (Methods), that is,

1 $$\begin{array}{rcl}{\omega }_{1,2}=\pm \frac{\sqrt{{\gamma }_{{\mathrm{EP}}}^{2}-{\gamma }^{2}}+\sqrt{{\gamma }_{{\mathrm{UP}}}^{2}-{\gamma }^{2}}}{2\sqrt{1+2c}}\times {\omega }_{0},\\ {\omega }_{3,4}=\pm \frac{\sqrt{{\gamma }_{{\mathrm{EP}}}^{2}-{\gamma }^{2}}-\sqrt{{\gamma }_{{\mathrm{UP}}}^{2}-{\gamma }^{2}}}{2\sqrt{1+2c}}\times {\omega }_{0}.\end{array}$$

Here ${\gamma }_{{\mathrm{EP}}}^{}=\sqrt{1+2c}-1$ denotes the exceptional point (EP)^11,12^ and ${\gamma }_{{\mathrm{UP}}}^{}=\sqrt{1+2c}+1$ is the upper critical point^23^; *c* is defined as the capacitive coupling ratio between the coupling capacitance *C*_C_ and the RLC resonator’s capacitance *C*; and *γ* is the normalized tuning parameter of gain (loss), defined as $\frac{\sqrt{L/C}}{R}$. In the unbroken phase ($0 < \frac{\gamma }{{\gamma }_{{\mathrm{EP}}}^{}} < 1$), the system is characterized by four purely real eigenfrequencies, with two of them positive (*ω*_1_, *ω*_3_) and the other two negative (*ω*_2_, *ω*_4_). In the broken phase ($\frac{\gamma }{{\gamma }_{{\mathrm{EP}}}^{}} > 1$, $\frac{\gamma }{{\gamma }_{{\mathrm{UP}}}^{}} < 1$), the eigenfrequencies are complex conjugate pairs with non-vanishing real parts. Above $\frac{\gamma }{{\gamma }_{{\mathrm{UP}}}^{}} > 1$, the eigenfrequencies become two complex conjugate pairs with purely imaginary parts.

**Fig. 2:**
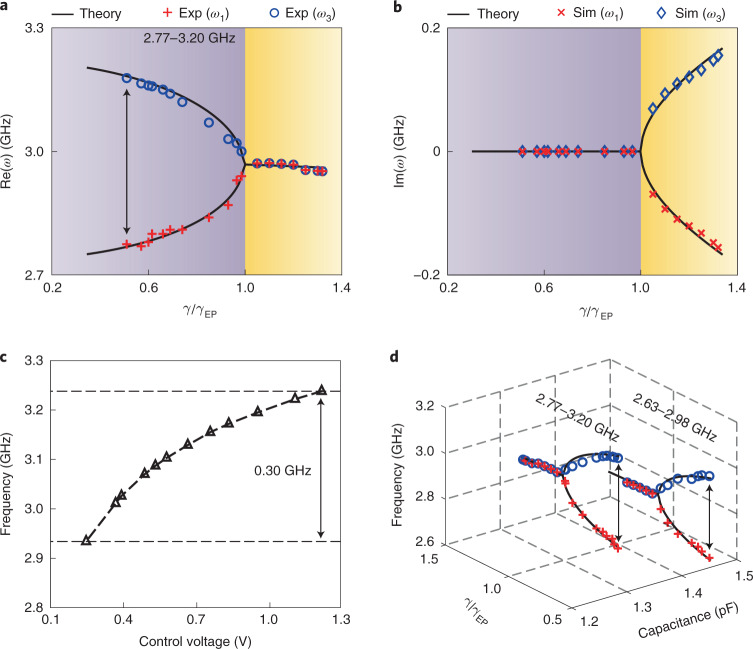
Eigenfrequencies and phase transition of the fully integrated PT-symmetric electronic system with the evolution of coupling factor $\frac{\gamma }{{\gamma }_{{\mathrm{EP}}}}$. **a**,**b**, Real (**a**) and imaginary (**b**) parts of eigenfrequencies as a function of the coupling factor $\frac{\gamma }{{\gamma }_{{\mathrm{EP}}}}$ when the resonators have a capacitance *C* = 1.30 pF. In both **a** and **b**, symbols correspond to experimental (Exp) or simulated (Sim) data, while the curves show theoretical results obtained with the small-signal model. Purple shading means the unbroken (exact) phase and yellow shading means the broken phase. **c**, FTR of the baseline oscillator, which is achieved by adjusting the varactor in the gain resonator through external voltage bias. The tuning range of the capacitance *C* is from 1.30 pF to 1.55 pF under different control voltages, corresponding to a FTR of 2.93–3.23 GHz, indicated by the dashed lines. **d**, FTR of our system at two fixed capacitances of resonators, *C* = 1.30 pF and *C* = 1.45 pF. By tuning the capacitance from 1.30 pF to 1.45 pF, the FTR changes from the interval of 2.77–3.20 GHz to the interval of 2.63–2.98 GHz, enabling a 2.63–3.20 GHz tuning range in total. The red and blue symbols are as in **a**. Source data

Experimentally, we engineered *γ* by adjusting the gain (loss) and maintaining other circuit parameters unchanged. The bifurcation of the eigenfrequencies in regards to the coupling factor $\frac{\gamma }{{\gamma }_{{\mathrm{EP}}}^{}}$ was clearly demonstrated (Fig. 2a). In the unbroken phase, the system has two purely real eigenfrequencies. We observed that the two RLC resonators had the same magnitude of voltage. When *γ* increases, the system undergoes a phase transition at the exceptional point, where the real eigenfrequencies branch out into the complex plane. In the broken phase, the system possesses two supermodes formed by the coupling of two RLC resonators. These two supermodes each have a single resonant frequency, one with amplification and the other with dissipation. The imaginary parts of the eigenfrequencies in the broken phase were obtained by Simulation Program with Integrated Circuits Emphasis (SPICE; simulation methods provided in Supplementary Section 5.2), since the oscilloscope used in our study could not capture the fast-changing dynamics of the exponentially oscillating amplitudes at the terminals (*V*_G_ and *V*_L_, where *V*_G_ (*V*_L_) represents the voltage amplitude at the gain (loss) side terminal as labelled in Fig. 1a) of the resonators. In the simulation, we observed an exponential growth (ending up at a saturation level) corresponding to the supermode with gain; the decay rates of the other supermode are the mirror of those for amplification (Fig. 2b). These results agree well with the theoretical predictions.

## Wide-band high-quality microwave generation

The resonant behaviour in the PT symmetry phase transition of our system provides a new strategy to generate microwave signals. Conventional microwave generation uses gain to fully compensate the intrinsic loss of inductor–capacitor (LC) resonators to generate a stable wave. PT symmetry provides a new degree of freedom to modulate microwave generation: by manipulating the gain–loss distribution in two coupled resonators, loss can play a role as important as that of gain. This unique gain–loss tuning freedom can enhance the bandwidth of microwave generation beyond that of conventional microwave generators (that is, oscillators based on a single-resonator structure or a coupled-resonator structure) with only a capacitive^40^ tuning or inductive^41^ tuning scheme. We theoretically compared the eigenfrequencies of all these systems (Supplementary Section 4). A PT-symmetric system inherently has a larger resonance tuning range given by equation (1), enabled by the eigenfrequencies’ bifurcation of tuning the gain–loss contrast *γ*. By comparison, the eigenfrequency of a conventional oscillator depends only on ${\omega }_{0}=\frac{1}{\sqrt{LC}}$, independent of gain–loss strength.

To experimentally show the advantages of a PT-symmetric system for microwave generation, we then decoupled the two RLC resonators via an on-chip switch (Fig. 1a). For a fair comparison, the active RLC resonator with a capacitive tuning was regarded as a baseline traditional oscillator (Methods). In the experiments, the baseline yielded a 0.30 GHz (2.93–3.23 GHz) bandwidth tuning by adjusting the control voltage of the varactor (Fig. 2c). By comparison, with the same amount of varactor tuning, the PT-symmetric system achieved a wider bandwidth tuning range of 0.57 GHz (2.63–3.20 GHz) by manipulating the gain–loss contrast (Fig. 2d), effectively enabling 2.1 times the frequency tuning range (FTR) of the baseline. We also observed that phase noise performances of our system were generally 1.5 dB better than the baseline across different oscillation frequencies (Supplementary Fig. 15), equivalent to 70% of the baseline noise intensity. This quality improvement can be attributed to the enhanced intrinsic passive quality factor in a PT-symmetric system and the subsequently increased carrier power through gain–loss tuning (Supplementary Section 4.2). These experiments demonstrate that PT symmetry can broaden the bandwidth tuning range of conventional microwave generation with improved quality. Our further investigations also show that our system generates four-phase microwaves using the unique topology at the exceptional point (Supplementary Fig. 16).

## Nonlinearity characterization and scattering properties

**Fig. 3:**
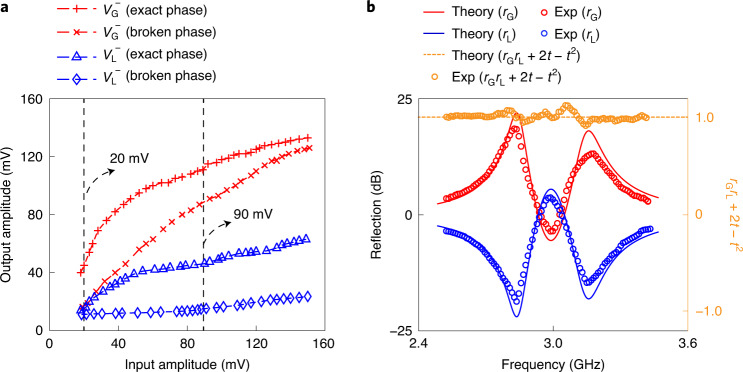
Nonlinearity characterization and generalized power conservation of the fully integrated PT-symmetric electronic system. **a**, Backward and forward input–output responses in both the exact phase and broken phase. Nonlinear responses occur in both regimes when the amplitudes of the input signals are high, but are stronger in the broken phase with a steeper slope. The black dashed lines mark the input amplitudes we chose to investigate the phenomenon of non-reciprocal transmission. **b**, Demonstrations of the generalized unitary relationship (also known as the generalized power conservation) of our system. We used two different methods to exhibit this property. First, we use decibels (dB; the left-hand *y* axis in the figure) to represent forward (*r*_G_) and backward (*r*_L_) reflection coefficients and show the comparison of *r*_G_, and *r*_L_ between experimental (Exp) measurements (empty circles) and PT symmetry theory (solid curves) in the linear region of the unbroken phase. The coefficients are symmetric about the 0 dB axis. Second, we demonstrate the quantity of *r*_G_ × *r*_L_ + 2*t* − *t*^2^ (the right-hand *y* axis in the figure). It shows that the experimental results (Exp) are much close to the PT symmetry theory (dashed curve with a constant value 1) across the spectrum. Note that in this experiment, we use a single-port^22^ set-up (Supplementary Section 2.1) for the scattering measurement, where the transmission *t* is 0. Both ways verify the property. Source data

Although a small-signal model is used to simplify the analysis, nonlinearities are prevalent in CMOS integrated systems when they operate at high frequency and in a large-signal domain. The XDP in our system is a nonlinear gain generator, which provides both a frequency-dependent^40^ and a complex high-order amplitude-dependent^42^ negative conductance (Supplementary Section 1.2), in contrast to the simple low-order amplitude-dependent gain intentionally introduced in previous PT-symmetric systems^21,43^. To experimentally characterize the nonlinear response of our system, the equivalent of a transmission line (TL)^22^ with characteristic impedance *Z*_0_ was attached to both sides of the system through an on-chip switch in the form of a resistor, *R*_0_ = *Z*_0_. We biased the system in either the unbroken or the broken phase and then monitored the output voltage ${V}_{{\mathrm{G}}}^{-}$ (${V}_{{\mathrm{L}}}^{-}$) at the gain (loss) side by sending the amplitude-varying input ${V}_{{\mathrm{L}}}^{+}$ (${V}_{{\mathrm{G}}}^{+}$) into the loss (gain) side (Fig. 1a). For a large input voltage (90 mV), a nonlinear response was clearly observed in both phases. For a small input voltage (20 mV), while a linear response was observed in the unbroken phase, a nonlinear response was clearly observed in the broken phase (Fig. 3a). This enhanced nonlinearity can be attributed to the field localization in the active RLC resonator^21^. Note that in this and the following experiments, we delicately tuned the gain and loss distribution to make our system operate in non-oscillatory mode while keeping the balanced PT symmetry condition.

Based on the characterization, we first studied the reflection of our system in the linear region. The scattering theory^22,44^ of linear PT-symmetric systems has shown that their reflection fulfils generalized unitary relationships; that is, the gain side reflection *r*_G_ and the loss side reflection *r*_L_ satisfy *r*_G_ × *r*_L_ = 1 across the spectrum (Supplementary Section 2.1). To experimentally demonstrate this property, a TL was connected at the gain or loss side (Fig. 1a), and the system was biased in the unbroken phase. Then, a sinusoidal signal with varied frequencies was applied into the system. The incident wave, ${V}_{{\mathrm{G}}}^{+}$ (${V}_{{\mathrm{L}}}^{+}$), and the reflected wave, ${V}_{{\mathrm{G}}}^{-}$ (${V}_{{\mathrm{L}}}^{-}$), were extracted from the voltages at either side of the TL, from which the reflection coefficients ${r}_{{\mathrm{G}}}={V}_{{\mathrm{G}}}^{-}/{V}_{{\mathrm{G}}}^{+}$ and ${r}_{{\mathrm{L}}}={V}_{{\mathrm{L}}}^{-}/{V}_{{\mathrm{L}}}^{+}$ were calculated. The measured reflections in Fig. 3b match well with the theoretical predictions. This scattering property has been demonstrated before for telemetry sensing^31^ by magnetically coupling two RLC resonators. Our system, built upon capacitive coupling, would also be promising as an integrated chemical sensor if the capacitors of the RLC resonators were devised similarly to those in previous works^45^. We also investigated the two-port scattering property^4–6^ (Supplementary Section 2.2) and observed the simultaneous existence of a coherent perfect absorption and lasing mode^4–6^ in this system (Supplementary Fig. 17). Moreover, extending our dimer system with more-complex PT-symmetric structures can produce more-advanced scattering phenomena, such as unidirectional invisibility^46,47^ (Supplementary Section 8.1).

## Magnetic-free non-reciprocal microwave transmission

PT-symmetric systems have demonstrated enhanced non-reciprocal acoustical and optical wave transmissions^14,15,21^ with introduced nonlinear effects. We next studied the non-reciprocal microwave transport of our system operating at different regions. We measured the forward and backward transmissions of our system at different coupling factors $\frac{\gamma }{{\gamma }_{{\mathrm{EP}}}}$ by tuning the gain–loss contrast. In these experiments, the same experimental set-up shown in Fig. 1a was adopted. A signal with variable frequencies was introduced into the gain (loss) side and captured at the loss (gain) side. By measuring the incident wave ${V}_{{\mathrm{G}}}^{+}$ (${V}_{{\mathrm{L}}}^{+}$) at the gain (loss) side and the transmitted wave ${V}_{{\mathrm{L}}}^{-}$ (${V}_{{\mathrm{G}}}^{-}$) at the corresponding loss (gain) side, both the forward transmission ${t}_{{\mathrm{F}}}={V}_{{\mathrm{L}}}^{-}/{V}_{{\mathrm{G}}}^{+}$ and backward transmission ${t}_{{\mathrm{B}}}={V}_{{\mathrm{G}}}^{-}/{V}_{{\mathrm{L}}}^{+}$ were obtained.

**Fig. 4:**
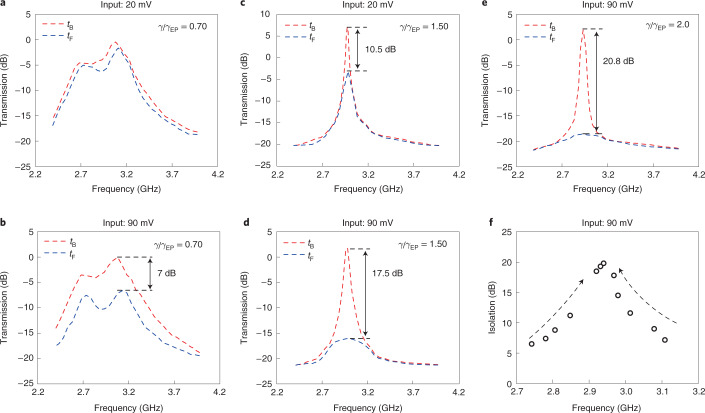
Non-reciprocal transmission of the fully integrated PT-symmetric electronic system. **a**, Reciprocal transmission in the unbroken phase ($\frac{\gamma }{{\gamma }_{{\mathrm{EP}}}}=0.70$) with a small input amplitude (20 mV). **b**, Non-reciprocal transmission in the unbroken phase ($\frac{\gamma }{{\gamma }_{{\mathrm{EP}}}}=0.70$) with increased input amplitude (90 mV). **c**, Non-reciprocal transmission in the broken phase ($\frac{\gamma }{{\gamma }_{{\mathrm{EP}}}}=1.50$) with a single peak at a small input amplitude (20 mV). **d**, Enhanced non-reciprocal transmission in the broken phase ($\frac{\gamma }{{\gamma }_{{\mathrm{EP}}}}=1.50$) with increased input amplitude (90 mV). **e**, Further enhanced non-reciprocal transmission in the broken phase with increased coupling factor ($\frac{\gamma }{{\gamma }_{{\mathrm{EP}}}}=2.0$) at the same input amplitude (90 mV). In **b**–**e**, the dashed lines indicate the peaks of the forward and backward transmission. The arrow between them shows the difference, that is, the isolation. **f**, The isolation *t*_B_ − *t*_F_ optimized by tuning the gain–loss parameter is shown at different probing frequencies in the microwave domain (2.75–3.10 GHz). The dashed arrows show the increasing trend of isolation. Source data

In the unbroken phase (Fig. 4a,b), the transmission spectra in both directions show double resonances. However, reciprocal transmission is observed with a 20 mV input amplitude, while non-reciprocal transmission is observed with a 90 mV input amplitude: the forward transmission goes up to −7 dB, and the backward transmission is 0.1 dB. In the low input power case, the system is linear and reaches an equilibrium between the two RLC resonators, giving rise to the similar microwave transmissions in both directions. Nonlinearity appears with increasing input amplitudes. The two resonators have different levels of nonlinearity, resulting in the non-reciprocal transmission. In the broken phase (Fig. 4c,d), the transmission spectra in both directions show single resonance. Non-reciprocal transmissions are observed under both input cases with different input amplitudes, and the case with the larger input voltage shows more notable non-reciprocal transmission. In addition, the comparison of the different phases under the same 90 mV input amplitude (Fig. 4b,d) shows that the nonlinearity is greatly enhanced in the broken phase, owing to the field localization in the active RLC resonator, and therefore the non-reciprocity is also enhanced: *t*_F_ decreases to −15.6 dB, and *t*_B_ slightly increases to 1.9 dB. Moreover, the non-reciprocity of the system is improved in the broken phase (Fig. 4e) under the same 90 mV input: *t*_F_ decreases to −18.7 dB, while *t*_B_ increases to 2.1 dB, due to the further enhanced nonlinearity, by increasing coupling factor $\frac{\gamma }{{\gamma }_{{\mathrm{EP}}}}$. By sweeping the coupling factor $\frac{\gamma }{{\gamma }_{{\mathrm{EP}}}}$ at a fixed 90 mV input amplitude (Supplementary Fig. 18), this non-reciprocal behaviour was observed over a broad bandwidth (2.75–3.10 GHz) at the resonance with the minimum isolation (Methods) over 7 dB (Fig. 4f).

Recent device demonstrations have produced non-magnetic non-reciprocity in silicon based on temporal modulation^33^, but often exhibit narrow bandwidths and have notable area overheads because a number of passive devices are required to perform complex modulations. Our system clearly demonstrates that PT symmetry with nonlinearity offers an approach to achieving broadband non-magnetic non-reciprocal transmissions by tuning the gain–loss contrast. Compared to state-of-the-art CMOS non-reciprocal devices^48^, our fully integrated PT-symmetric electronic system shows strong isolation (7–21 dB) among a wider microwave (2.75–3.10 GHz) bandwidth without complex modulations that require a huge and expensive on-chip area. Our system also shows strong non-reciprocity with a lower input power threshold (−21 dBm) compared to nonlinearity-induced non-reciprocal devices on other electronic platforms^21,38^ (Supplementary Table 2). This excellent performance could lay the foundation for abundant application advancements in quantum computing^33^, device protection^38,49^ and radar communication^50^.

## Conclusions

We have reported a fully integrated electronic platform based on a CMOS process technology for non-Hermitian physics, validating the powerful role of IC to study PT symmetry in a scalable manner. Fully integrated PT-symmetric electronics enables capabilities in the microwave domain not seen in previous electronic platforms^22–31^, to the best of our knowledge. With the unique gain–loss tuning mechanism of PT symmetry, our system shows extended broadband response and improved noise performance for microwave generation over conventional devices. Moreover, our chip demonstrates strong non-reciprocal microwave transmission with the enhanced intrinsic nonlinearity of IC, leading to a generation of integrated non-magnetic non-reciprocal devices. Our results shed light on PT symmetry as an innovative design approach to overcoming the limitations of IC performances and benefiting numerous applications. In addition, more-advanced IC technologies can be used to extend the functional and performance benefit of PT-symmetric systems to the higher millimetre wave and terahertz frequency range. The study is also expected to motivate further exploration such as PT symmetry in optoelectronics^18^, electro-acoustics^21^ and topological electronics (Supplementary Section 8.2) based on high-dimensional PT-symmetric structures^28–30^ with standard IC technology, enriching the scientific discoveries of non-Hermitian physics.

## Methods

### Differential architecture

The fully integrated PT-symmetric electronic system was implemented in a differential topology (Supplementary Fig. 1). The gain −*R*_G_ is the parallel resistance of −*R*_G0_, −*R*_G1_ and *R*_G2_. The −*R*_G0_ is generated by the XDP. The *R*_G1_ is realized by a voltage-controlled metal–oxide–semiconductor (MOS) resistor. The loss *R*_L0_ is realized in the same way as *R*_G1_. By fixing the bias voltage *V*_BIASG_ of −*R*_G0_ and controlling the bias voltage of the gain (loss) side MOS resistor, −*R*_G_ (*R*_L_) can be continuously adjusted. The capacitor *C*_G_ (*C*_L_) in each RLC resonator is composed of a MIM capacitor *C*_G1_ (*C*_L1_) and a varactor *C*_G2_ (*C*_L2_). The varactor takes up only a small proportion of the total capacitance in each RLC resonator and is used to compensate the mismatch between the fixed MIM capacitor on both sides. The coupling capacitance *C*_C_ is designed by two equal MIM capacitors *C*_C1_ and *C*_C2_, which are serially connected through an on-chip switch (Fig. 1a). The inductance *L*_G_ (*L*_L_) in both RLC resonators comes from symmetrical parallel inductors (Supplementary Fig. 2). In the practical implementation, the equivalent of a TL with characteristic impedance *Z*_0_ was attached to each side of the system through an on-chip switch in the form of an on-chip resistor *R*_0_ = *Z*_0_. By controlling the switch, the system could be flexibly configured to test one-port scattering or non-reciprocal transmission.

In the differential architecture, each signal is transmitted by a pair of differential wires, where the signal is represented by the amplitude difference between the differential wires. For example, in our design, each voltage *V*_G_ (*V*_L_) at the terminal of the RLC resonator is represented by a pair of differential signals (*V*_GP_ and *V*_GN_ for *V*_G_; *V*_LP_ and *V*_LN_ for *V*_L_). *V*_LP_ and *V*_LN_ (*V*_GP_ and *V*_GN_) are one pair of differential signals labelled in the differential structure of our system as shown in Supplementary Fig. 1, which correspond to the signal-end signal *V*_L_ (*V*_G_) labelled in Fig. 1a. The differential architecture is symmetric with respect to its virtual ground and can be divided into two equal parts (Supplementary Fig. 3). Either of them is an equivalent single-ended representation of the differential one and can be used to derive the PT symmetry concept. Note that in a single-ended architecture, the gain −*R*_G_, loss *R*_L_ and inductor *L*_G_ (*L*_L_) will be half, and the capacitor *C*_G_ (*C*_L_) will be double. The PT symmetry condition is satisfied by setting *R*_G_ ≈ *R*_L_ = *R*, *L*_G_ ≈ *L*_L_ = *L* and *C*_G_ ≈ *C*_L_ = *C*. In our system, *R* ∈ (90, 380) Ω, *L* = 1.85 nH, *C* ∈ (1,300, 1,550) fF, *C*_C_ = 500 fF and *Z*_0_ = 280 Ω.

### Phase transition

Applying Kirchoff’s law on the equivalent circuit representation in Fig. 1a yields the following expression^22^:

2 $${V}_{{\mathrm{G}}}={\mathrm{i}}{\omega }^{\prime}L/2\times {I}_{1},\,\quad {I}_{1}-{V}_{{\mathrm{G}}}/(R/2)+{\mathrm{i}}{\omega }^{\prime}2C {V}_{{\mathrm{G}}}+{\mathrm{i}}{\omega }^{\prime}{C}_{{\mathrm{C}}} ({V}_{{\mathrm{G}}}-{V}_{{\mathrm{L}}})=0,$$

3 $${V}_{{\mathrm{L}}}={\mathrm{i}}{\omega }^{\prime}L/2\times {I}_{2},\,\quad {I}_{2}+{V}_{{\mathrm{L}}}/(R/2)+{\mathrm{i}}{\omega }^{\prime}2C{V}_{{\mathrm{L}}}+{\mathrm{i}}{\omega }^{\prime}{C}_{{\mathrm{C}}} ({V}_{{\mathrm{L}}}-{V}_{{\mathrm{G}}})=0.$$

Here *ω*′ is an angular frequency. *I*_1_ (*I*_2_) denotes the current flowing into the inductor on the gain (loss) side. Eliminating the current from the relationships, scaling the frequency and time by ${\omega }_{0}=\frac{1}{\sqrt{LC}}$ and taking *c* = *C*_C_/2*C*, $\gamma =\sqrt{L/C}/R$ gives the following matrix equation^22^:

4 $$\left[\begin{array}{ll}1/\omega -{\mathrm{j}}\gamma -\omega (1+c)&\omega c\\ \omega c&1/\omega +{\mathrm{j}}\gamma -\omega (1+c)\\ \end{array}\right]\cdot \left[\begin{array}{l}{V}_{{\mathrm{G}}}\\ {V}_{{\mathrm{L}}}\end{array}\right]=\left[\begin{array}{l}0\\ 0\end{array}\right].$$

Here *ω* is the normalized frequency and j is the unit imaginary number. This linear, homogeneous system has four normal mode frequencies, as required to fulfil any arbitrary initial condition for voltage and current, given by^22^

5 $${\omega }_{1,2}=\pm \frac{\sqrt{{\gamma }_{{\mathrm{EP}}}^{2}-{\gamma }^{2}}+\sqrt{{\gamma }_{{\mathrm{UP}}}^{2}-{\gamma }^{2}}}{2\sqrt{1+2c}},\,\quad {\omega }_{3,4}=\pm \frac{\sqrt{{\gamma }_{{\mathrm{EP}}}^{2}-{\gamma }^{2}}-\sqrt{{\gamma }_{{\mathrm{UP}}}^{2}-{\gamma }^{2}}}{2\sqrt{1+2c}},$$

where the PT-symmetric breaking point (*γ*_EP_) and the upper critical point (*γ*_UP_) are identified as ${\gamma }_{{\mathrm{EP}}}^{}=| 1-\sqrt{1+2c}|$ and ${\gamma }_{{\mathrm{UP}}}^{}=1+\sqrt{1+2c}$. The corresponding phase difference^22^ between the two RLC resonators can be expressed as

6 $${\phi }_{1,3}=\frac{\uppi }{2}-{\tan }^{-1}\left[\frac{1}{\gamma }\left(\frac{1}{{\omega }_{1,3}}-(1+c){\omega }_{1,3}\right)\right].$$

### FTR of microwave generation

The FTR (as a percentage) for microwave generation is defined as

7 $${\mathrm{FTR}}=\frac{{\omega }_{\max }-{\omega }_{\min }}{({\omega }_{\max }+{\omega }_{\min })/2}\times 100.$$

Here *ω*_max_ and *ω*_min_ are the maximum and minimum frequencies, respectively, in the total tuning bandwidth.

### Isolation of non-reciprocal transmission

The isolation of our system under a coupling factor $\frac{\gamma }{{\gamma }_{{\mathrm{EP}}}}$ is defined as

8 $${I}_{{\mathrm{ISO}}}=\max ({t}_{{\mathrm{B}}}-{t}_{{\mathrm{F}}}).$$

Here *t*_B_ and *t*_F_ are the backward transmission and forward transmission of the system with the frequency sweeping.

### Chip implementation and fabrication

All CMOS devices were prepared in Cadence Virtuoso (an industry-standard design tool for front-end circuit design). All designs satisfy the standard CMOS manufacturing rules of IBM’s commercial 130 nm CMOS8RF process, with physical verification performed using Mentor Graphics Calibre (an industry-standard design tool for back-end layout design). We used Metal Oxide Semiconductor Implementation Service (MOSIS) for fabrication.

### Measurement set-ups

The chip was bonded on a four-layer FR4 PCB (used as a daughterboard in our experiments) by gold wires (Supplementary Fig. 12), forming all 38 electrical connections (including power and ground) from the chip to the PCB. The bonding wires were properly designed for use at frequencies above 50 GHz. Our experimental set-up comprised a bonded chip in a daughterboard, a motherboard, a power supply, a mixed signal oscilloscope (MSO; Agilent 9404A), an arbitrary wave generator (AWG; KEYSIGHT M8195A) and a personal computer. The daughterboard provided control biases to the chip. These biases had two main functions: (1) compensating the mismatch of CMOS components to minimize the unbalance between the two RLC resonators, and (2) tuning the gain (loss) such that the system could evolve from the exact phase to the broken phase. The motherboard acted as a power board to supply all power/control voltages to the daughterboard. The benchtop power supply was the main power source and was used to power the motherboard. The MSO had four pairs of differential channels, and its highest sampling rate was 20 giga samplings per second. The AWG had four pairs of differential channels, and each pair could generate arbitrary waves of up to 50 GHz with independently varying phase. In the phase transition experiments, the outputs of two RLC resonators were connected to the MSO, where both the eigenfrequencies and phase differences could be directly observed on the panel. In the scattering experiments, the AWG sourced sinusoidal signals with varying frequencies or phase into the chip through the TL. Then signals on both terminals of the TL were sent into the MSO such that the incident wave and reflected wave could be captured. In the non-reciprocal experiments, the AWG fed sinusoidal signals with varying frequencies into the system through the gain (loss) side TL. Then both the incident wave on the input terminal of the gain (loss) side TL and the reflected wave on the output terminal of the loss (gain) side TL could be captured by the MSO. During the scattering experiments and non-reciprocal transport experiments, the AWG was controlled by software on a personal computer to generate frequency-varying signals.

## Online content

Any methods, additional references, Nature Research reporting summaries, source data, extended data, supplementary information, acknowledgements, peer review information; details of author contributions and competing interests; and statements of data and code availability are available at 10.1038/s41565-021-01038-4.

## Supplementary information

Supplementary Information Supplementary Figs. 1–23, Discussions 1–8 and Tables 1 and 2.

## Data Availability

The data that support the findings of this study are available within the article and its Supplementary Information. Source data are provided with this paper. Additional data are available from the corresponding authors upon request.

## Source data

Source Data Fig. 2 Statistical source data for figure plot.
Source Data Fig. 3 Statistical source data for figure plot.
Source Data Fig. 4 Statistical source data for figure plot.
