# Supplementary material for: Fully integrated parity–time-symmetric electronics
Source: Nat Nanotechnol. 2022 Mar 17;17(3):262–8. doi: 10.1038/s41565-021-01038-4 (PMC8930767; doi:10.1038/s41565-021-01038-4)
Supplement: Supplementary file 1 — Supplementary Figs. 1–23, Discussions 1–8 and Tables 1 and 2. [file 41565_2021_1038_MOESM1_ESM.pdf]

nature portfolio

https://doi.org/10.1038/s41565-021-01038-4

Supplementary information

Fully integrated parity–time-symmetric electronics

In the format provided by the authors and unedited

Supplementary Information of Fully Integrated Parity-Time-Symmetric Electronics

Weidong Cao[1,7], Changqing Wang[1], Weijian Chen[1,2,3], Song Hu[4,6], Hua Wang[4,5], Lan Yang[1,7], and Xuan Zhang[1,7]

[1]Department of Electrical and Systems Engineering, Washington University, St. Louis, MO, USA.

[2]Department of Physics, Washington University, St Louis, MO, USA.

[3]Center for Quantum Sensors, Washington University, St Louis, MO, USA.

[4]School of Electrical and Computer Engineering, Georgia Institute of Technology, Atlanta, GA, USA.

[5]Department of Information Technology and Electrical Engineering, Swiss Federal Institute of Technology Zurich, Zurich, Switzerland.

[6]Present address: Apple Inc, 1 Apple Park Way, Cupertino, CA, USA.

[7]Email: Corresponding authors: weidong.cao@wustl.edu; yang@seas.wustl.edu; xuan.zhang@wustl.edu.

1 Implementation

1.1 Differential Architecture And Detailed Circuits

The schematic overview of the proposed fully integrated parity-time- (PT-) symmetric electronic system is illustrated in Supplementary Figure 1. As it shows, our system was implemented with a differential topology, in contrast with the single-ended architecture commonly used in board-level [1] and MEMs-level [2] PT-symmetric electronic systems. The differential architecture has the advantage to mitigate common-mode perturbations.

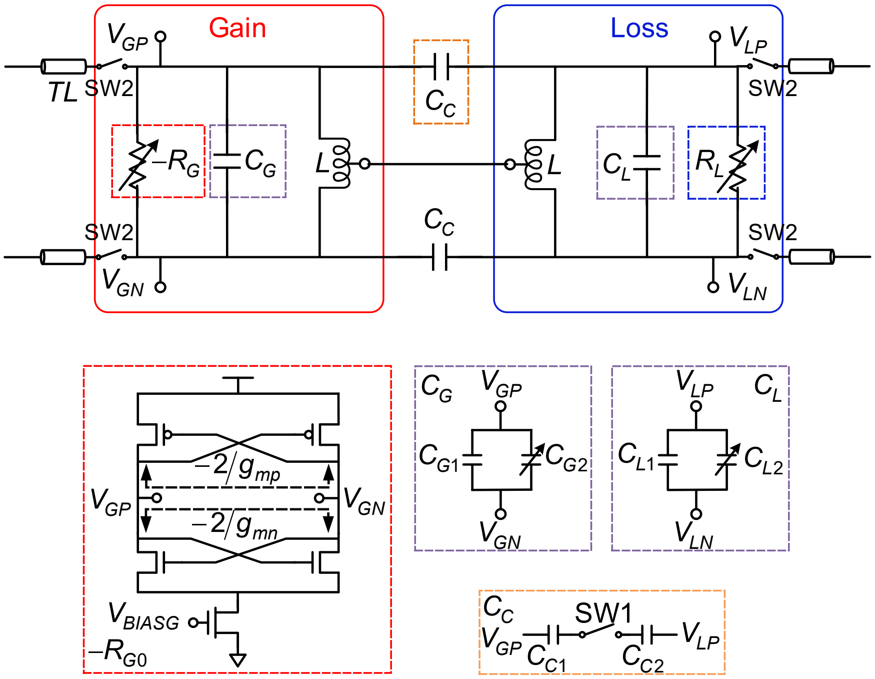

Supplementary Figure 1: Schematic overview of fully integrated PT-symmetric electronic system with differential architecture. It consists of two RLC resonators with balanced gain $-R_G$ and loss R_L. The gain $-R_{G0}$ is generated by the cross-coupled differential pair (XDP). The capacitances C_G (C_L) in both RLC resonators are comprised of an inherent parasitic capacitance C_{G0} (C_{L0}), a fixed high-Q MIM capacitor C_{G1} (C_{L1}) and a varactor C_{G2} (C_{L2}). The varactor is used to compensate the mismatch between C_{G1} and C_{L1}. The coupling capacitance (C_C) is made of two serially connected MIM capacitors (C_{C1} and C_{C2}) with an on-chip switch (SW1). The two RLC resonators can be coupled (decoupled) by turning on (off) the SW1. TLs are attached to the both sides of system by SW2.

Our system consists of two RLC resonators, one with active gain $-R_G$ and the other one with passive loss R_L. The gain $-R_G$ is the parallel resistance of $-R_{G0}$, R_{G1}, and R_{G2}, namely, $-R_G = -R_{G0}||R_{G1}||R_{G2}$. Here, $-R_{G0}$ is generated by the cross-coupled differential pair (XDP); R_{G1} is a variable resistor realized by MOS transistors [3, 4]; R_{G2} is the inherent loss of the active RLC resonator. Similarly, the loss R_L is the parallel resistance of R_{L0}, and R_{L1}, that is $R_L = R_{L0}||R_{L1}$. R_{L0} is a variable resistor realized by in the same way as R_{G1}; R_{L1} is the inherent loss of the passive RLC resonator. By controlling the bias voltage of gain (loss) side MOS transistors [3, 4], $-R_G$

(R_L) can be continuously adjusted. The capacitor C_G (C_L) in each RLC resonator is composed of a parasitic capacitance C_{G0} (C_{L0}), a fixed Metal-Insulator-Metal (MIM) capacitor C_{G1} (C_{L1}) with high-quality factor (high-Q) and an adjustable varactor C_{G2} (C_{L2}). The varactor takes up a small proportion of the total capacitance and is used to compensate for the fabricated mismatch between the fixed MIM capacitors of both sides. The coupling capacitance C_C is designed by two equal MIM capacitors C_{C1} and (C_{C2}) in serial connection via an on-chip switch (SW1). Note that the two RLC resonators can also be coupled (decoupled) by turning on (off) of the SW1.

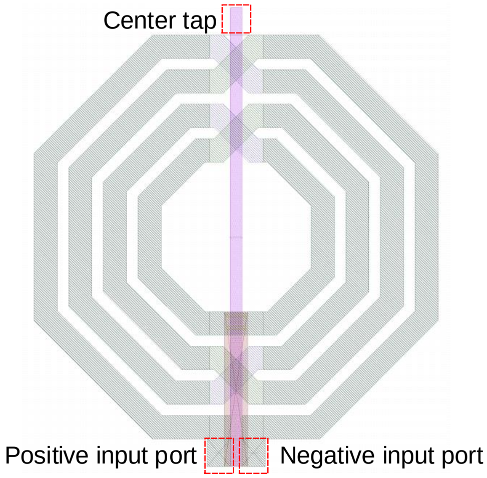

Supplementary Figure 2: Symmetrical physical layout of symindp. It has a pair of differential input ports and a center tap.

The inductor L in each RLC resonator is a symmetrical parallel inductor (symindp) with three terminals: two input ports and one center tap. The center tap connection is provided such that by connecting the center taps of inductors in both RLC resonators, the passive RLC resonator shares the same common-mode voltage with the active one. This symindp with cross-over connections to create a symmetrical layout (Supplementary Figure 2) with low resistance and low parasitic capacitance, is ideally suited for differential resonators. In the practical implementation, the equivalent of a transmission line (TL) with characteristic impedance Z_0 was attached to both sides of the system through an on-chip switch (SW2) in the form of a resistor $R_0 = Z_0$.

A common way to analyze differential circuits is to convert them into single-ended equivalents. As the Supplementary Figure 3a shows, by using a symmetrical axis[1], the differential circuit can be divided into two equal parts. Either of them is an equivalent single-ended representation of the differential one, and can be used to derive mathematical expression of the PT-symmetric system. Therefore, the equivalent single-ended representation in Supplementary Figure 3b is used as the simplified model for analysis through our paper. The difference between these two circuit topologies

[1]The horizontal dashed yellow line shown in Supplementary Figure 3a.

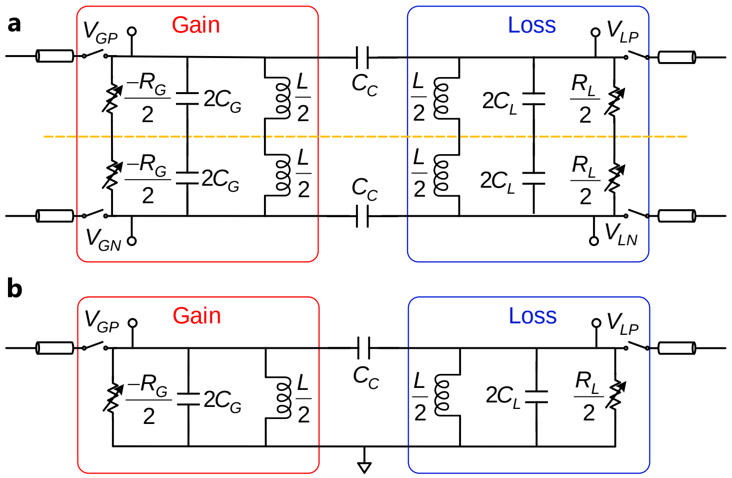

Supplementary Figure 3: Transformation of differential architecture to equivalent single-ended architecture. a. The symmetric representation of PT-symmetric electronic system. The yellow dash line is a symmetric axis, also as known as virtual ground. **b**. The equivalent single-ended circuit schematic of PT-symmetric electronic system.

is that in differential architecture, each signal is represented by the amplitude difference between the differential wires; while in single-ended architecture, each signal is transmitted by only one wire and all the terminal signals are referenced to a common ground. For example, in our system, the voltage V_G (V_L) at the terminal of each RLC resonator is represented by a pair of differential signals, e.g., V_{GP} and V_{GN} for V_G, V_{LP} and V_{LN} for V_L. Note that in single-ended architecture, gain $-R_G$, loss R_L and inductor L will be half, but the capacitor C_G (C_L) will be double. The PT symmetry condition is satisfied by setting $R_G \approx R_L = R$, $L_G \approx L_L = L$, and $C_G \approx C_L = C$.

1.2 Analysis of Cross-coupled Differential Pair

A comprehensive analysis of $-R_{G0}$ can be obtained through the small signal model [5] of XDP shown in Supplementary Figure 4, where for NMOS differential pair we have

$$Y_{X,n}(S) = \frac{R_{G,n}C_{GS,n}C_{GD,n}S^2 + [C_{GS,n} + (4 + g_{m,n}R_{G,n})C_{GD,n}] - g_{m,n}}{2[R_{G,n}(C_{GS,n} + C_{GD,n}) + 1]}, \tag{1}$$

and for PMOS differential pair,

$$Y_{X,p}(S) = \frac{R_{G,p}C_{GS,p}C_{GD,p}S^2 + [C_{GS,p} + (4 + g_{m,p}R_{G,p})C_{GD,p}] - g_{m,p}}{2[R_{G,p}(C_{GS,p} + C_{GD,p}) + 1]}. \tag{2}$$

Hence,

$$Re\{Y_{X,n}\} = \frac{-g_{m,n} + R_{G,n}\omega^2[C_{GS,n}^2 + C_{GD,n}(4 + g_{m,n}R_{G,n})(C_{GS,n} + C_{GD,n})]}{2[R_{G,n}^2(C_{GS,n} + C_{GD,n})^2\omega^2 + 1]}, \tag{3}$$

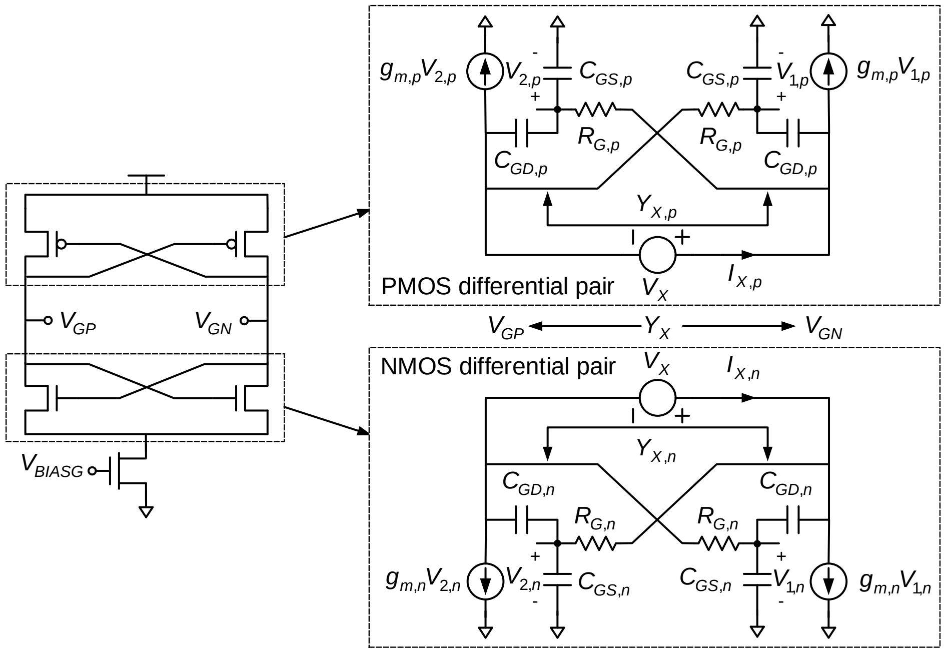

Supplementary Figure 4: Small signal model of cross-coupled differential pair. This model considers parasitic effect (gate resistor R_G, gate-drain capacitance C_{GD}, gate-source capacitance C_{GS}) when the circuit operates at high frequency.

$$Re\{Y_{X,p}\} = \frac{-g_{m,p} + R_{G,p}\omega^2[C_{GS,p}^2 + C_{GD,p}(4 + g_{m,p}R_{G,p})(C_{GS,p} + C_{GD,p})]}{2[R_{G,p}^2(C_{GS,p} + C_{GD,p})^2\omega^2 + 1]}. \tag{4}$$

Here, the subscript n (p) denotes NMOS (PMOS) differential pair. If $R_{G,n}^2(C_{GS,n} + C_{GD,n})^2\omega^2 \ll 1$ and $R_{G,p}^2(C_{GS,p} + C_{GD,p})^2\omega^2 \ll 1$, then

$$Re\{Y_{X,n}\} \approx -\frac{g_{m,n}}{2} + R_{G,n}\omega^2 \cdot \frac{C_{GS,n}^2 + C_{GD,n}(4 + g_{m,n}R_{G,n})(C_{GS,n} + C_{GD,n})}{2}, \tag{5}$$

$$Re\{Y_{X,p}\} \approx -\frac{g_{m,p}}{2} + R_{G,p}\omega^2 \cdot \frac{C_{GS,p}^2 + C_{GD,p}(4 + g_{m,p}R_{G,p})(C_{GS,p} + C_{GD,p})}{2}. \tag{6}$$

Combing Eq. (5) and Eq. (6), one can obtain

$$Re\{Y_X\} = Re\{Y_{X,n}\} + Re\{Y_{X,p}\} = -(g_{m,n} + g_{m,p})/2 + f(\omega). \tag{7}$$

Here, $f(\omega)$ is a frequency-dependent term. In large signal domain, $Re\{Y_X\}$ also dependents on the amplitude of oscillation voltage between the two terminals of the differential pair [6]. A reasonable assumption is that when frequency ω is low and the XDP operates in the small signal domain, $Re\{Y_X\}$ can be expressed as

$$Re\{Y_X\} = -(g_{m,n} + g_{m,p})/2. \tag{8}$$

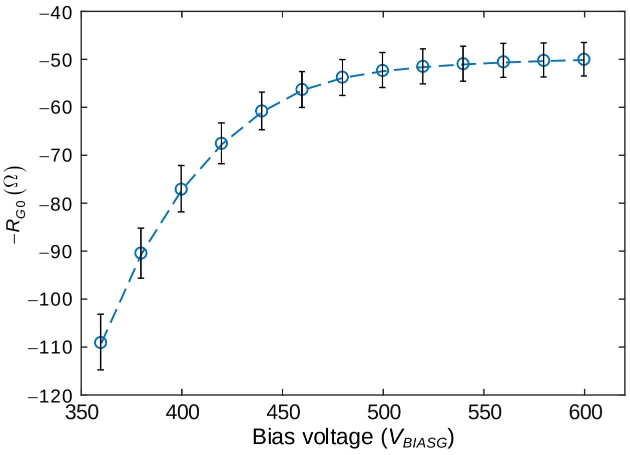

Supplementary Figure 5: Tuning gain with the bias voltage. The results are obtained from the thorough post-layout simulation with the consideration of PVT variations.

Therefore, the negative resistance $-R_{G0}$ can be obtained as

$$-R_{G0} = -2/(g_{m,n} + g_{m,p}), \tag{9}$$

which is the reciprocal of summation of small signal transconductance of NMOS differential pair and PMOS differential pair. For theoretical analysis, we consider our system as a linear system with the assumption of small signal condition. Supplementary Figure 5 shows the dependence of the negative resistance on the bias voltage. The results are obtained from high-fidelity post-layout circuit simulations, where process, voltage, and temperature (PVT) variations are carefully considered. The error bar at each point is the potential variation range of the negative resistance caused by PVT variations at the same bias voltage. Note that for analog circuit design in mature technology (such as CMOS 130 nm), the simulated results from high-fidelity post-layout simulator (i.e., Cadence Spectre) often match well with the measured results, and thus can be used to verify the functionalities of the designed chip.

2 Scattering Properties

2.1 Single-port Scattering

The theory of linear PT-symmetric systems has shown that single-port scattering fulfills generalized unitary relationship, that is gain side reflection r_G and loss side reflection r_L satisfy $r_G \cdot r_L = 1$. We first derive the theory of single-port scattering for our system from the circuit perspective. Supplementary Figure 6 represents the conceptual illustration of single-port scattering and its equivalent circuit model. In scattering theory, the reflection coefficients are defined as the ratio of reflected

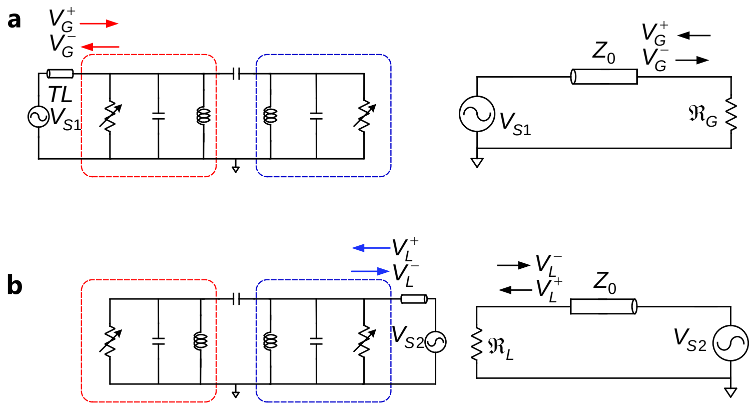

Supplementary Figure 6: Simplified model for single-port scattering associated with the fully integrated PT-symmetric electronic system. a. TL is attached to the gain side of the system. **b**. TL is connected to the loss side of the system.

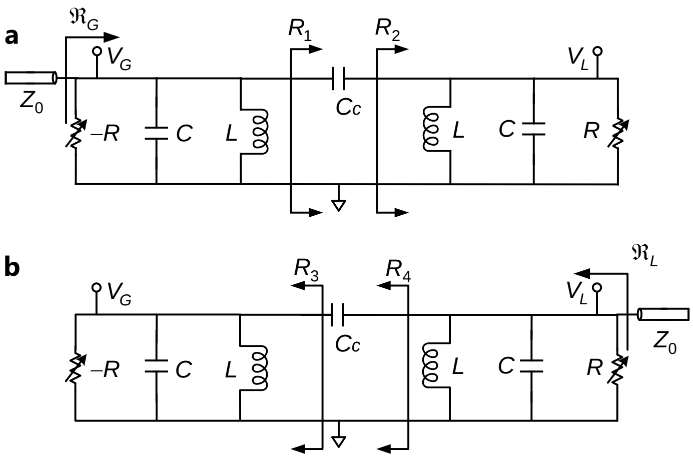

Supplementary Figure 7: Circuit models to calculate $\mathcal{R}_G$ and $\mathcal{R}_L$. a. Circuit model for calculating $\mathcal{R}_G$. **b**. Circuit model for calculating $\mathcal{R}_L$.

wave and incident wave, that is $r_G = V_G^- / V_G^+$ and $r_L = V_L^- / V_L^+$. In circuit theory, the gain side reflection r_G can be readily obtained as

$$r_G = \frac{\mathcal{R}_G(\omega') - Z_0}{\mathcal{R}_G(\omega') + Z_0}. \tag{10}$$

While for the loss side, the reflection r_L can be directly written as

$$r_L = \frac{\mathcal{R}_L(\omega') - Z_0}{\mathcal{R}_L(\omega') + Z_0}. \tag{11}$$

Here, $\mathcal{R}_G$ represents the equivalent impedance of the system seen from left to right (shown in Supplementary Figure 7a); $\mathcal{R}_L$ represents the impedance of the system seen from right to the left (shown in Supplementary Figure 7b); and Z_0 is the characteristic resistor of TL. Let $\gamma =$

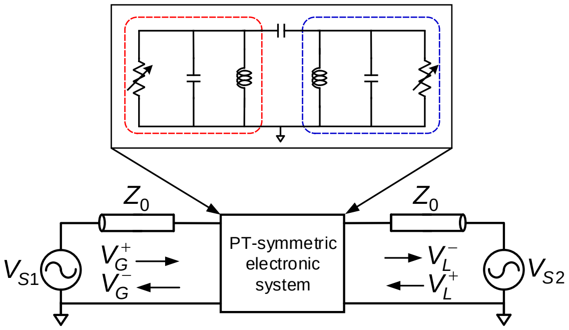

Supplementary Figure 8: Circuit models to calculate the total output coefficient. The upper is the PT-symmetric electronic system. The below is the circuit model.

$\sqrt{(L/C)}/R$, $c = C_C/C$, $\omega = \omega' \sqrt{LC}$, and resort to the Supplementary Figure 7a, then

$$\mathcal{R}_G = R \cdot \frac{-\omega^2\gamma^2 + j[\omega\gamma - (1+c)\omega^3\gamma]}{(1-\omega^2)^2 - 2\omega^2 c(1-\omega^2) + \omega^2\gamma^2}. \tag{12}$$

Similarly, we resort to the Supplementary Figure 7b to calculate $\mathcal{R}_L$ which is expressed as

$$\mathcal{R}_L = R \cdot \frac{\omega^2\gamma^2 + j[\omega\gamma - (1+c)\omega^3\gamma]}{(1-\omega^2)^2 - 2\omega^2 c(1-\omega^2) + \omega^2\gamma^2}. \tag{13}$$

Let $E = R\omega^2\gamma^2$; $F = R[\omega\gamma - (1+c)\omega^3\gamma]$; $G = (1-\omega^2)^2 - 2\omega^2 c(1-\omega^2) + \omega^2\gamma^2$, then

$$\mathcal{R}_G = (-E + jF)/G; \quad \mathcal{R}_L = (E + jF)/G. \tag{14}$$

By combining Eq. (10), Eq. (11), and Eq. (14), we can obtain

$$||r_G|| \cdot ||r_L|| = 1; \quad \phi_G + \phi_L = \pi. \tag{15}$$

2.2 Two-port Scattering

The theory of linear PT-symmetric systems has also shown that two-port scattering exhibits a simultaneous coherent perfect absorber (CPA-) -amplifier property at a special frequency (Janus frequency). We then derive the two-port scattering theory for our system from the circuit perspective. The two-port scattering can be considered as two-port TL model shown in Supplementary Figure 8. We can write the basic voltage law of system by using S-parameter model [1]:

$$\begin{cases} \frac{V_G^-}{\sqrt{Z_0}} = S_{11} \cdot \frac{V_G^+}{\sqrt{Z_0}} + S_{12} \cdot \frac{V_L^+}{\sqrt{Z_0}}; \\ \frac{V_L^-}{\sqrt{Z_0}} = S_{21} \cdot \frac{V_G^+}{\sqrt{Z_0}} + S_{22} \cdot \frac{V_L^+}{\sqrt{Z_0}}. \end{cases} \tag{16}$$

Here, in our system,

$$S = 1/(A - iB) \cdot \begin{bmatrix} -iD & 2\omega c\eta \\ 2\omega c\eta & iC \end{bmatrix}. \tag{17}$$

And,

$$\begin{cases} A = 2\eta\Omega; \\ B = \Omega^2 - \eta^2 - \omega^2 c^2 + \gamma^2; \\ C = (\gamma - \eta)^2 + \Omega^2 - \omega^2 c^2; \\ D = (\gamma + \eta)^2 + \Omega^2 - \omega^2 c^2; \\ \Omega = \omega(1 + c) - 1/\omega; \\ \gamma = \sqrt{L/C}/R; \\ \eta = \sqrt{L/C}/Z_0; \\ c = C_C/C; \\ \omega = \omega' \sqrt{LC}. \end{cases} \tag{18}$$

We can transform Eq. (16) into the following equation [1]:

$$\begin{bmatrix} V_L^- \\ V_L^+ \end{bmatrix} = \mathcal{M} \cdot \begin{bmatrix} V_G^- \\ V_G^+ \end{bmatrix}. \tag{19}$$

Here,

$$\mathcal{M} = \frac{1}{2\omega c\eta} \cdot \begin{bmatrix} A + iB & iC \\ -iD & A - iB \end{bmatrix}. \tag{20}$$

Note that $det(\mathcal{M}) = 1$. Therefore,

$$S = \frac{1}{\mathcal{M}_{22}} \cdot \begin{bmatrix} -\mathcal{M}_{21} & 1 \\ 1 & \mathcal{M}_{12} \end{bmatrix}. \tag{21}$$

Generally, the reflection and transmission coefficients for the gain (G) and loss (L) incidence in terms of the transfer matrix elements as [1]

$$r_G = -\frac{\mathcal{M}_{21}}{\mathcal{M}_{22}}, \quad r_L = -\frac{\mathcal{M}_{12}}{\mathcal{M}_{22}}, \quad t_G = t_L = \frac{1}{\mathcal{M}_{22}}. \tag{22}$$

It can be derived that

$$\begin{cases} ||r_G|| \cdot ||r_L|| = \sqrt{||(\mathcal{M}_{21} \cdot \mathcal{M}_{12})/(\mathcal{M}_{22} \cdot \mathcal{M}_{22})||} = \sqrt{||T - 1||}; \\ \phi_G + \phi_L = \pi. \end{cases} \tag{23}$$

Here, transmittance $T = t_G \cdot t_L$. In the single-port scattering case, the transmittance $T = 0$, as $\mathcal{M}_{22} \to \infty$ when $\eta \to 0$. In other words, $||r_G|| \cdot ||r_L|| = 1$. Therefore, Eq. (15) is a special case of Eq. (23).

Using the scattering matrix, one can derive the conditions that our PT-symmetric system can simultaneously act either as an amplifier or as a perfect absorber [1, 7, 8, 9, 10]. For a laser oscillator without an injected signal, the boundary condition satisfies $V_G^+ = V_L^+ = 0$, which indicates $\mathcal{M}_{22}(\omega) = 0$ in Eq. (21). For a perfect absorber, the boundary condition satisfies $V_G^- = V_L^- = 0$, which implies $det(S) = 0$ in Eq. (21). Therefore, $\mathcal{M}_{11}(\omega) = (1 + \mathcal{M}_{12}\mathcal{M}_{21})/\mathcal{M}_{22} = 0$, and the amplitudes of the incident waves must satisfy the condition $V_L^+ = \mathcal{M}_{21}(\omega)V_G^+$. For the PT-symmetric structure, the matrix elements of $\mathcal{M}$ in Eq. (20) satisfy the relationship $\mathcal{M}_{11}(\omega) = \mathcal{M}_{22}^*(\omega^*)$. Thus, a real $\omega = \omega_J$ (Janus frequency) exists, that satisfies the amplifier/laser condition simultaneously with the absorber condition ($\mathcal{M}_{11}(\omega_J) = \mathcal{M}_{22}(\omega_J) = 0$) [1, 7]. Hence the two-port PT-symmetric system can behave simultaneously as a perfect absorber and as an amplifier.

This property can be explored using an overall output coefficient Θ defined as [1, 7]

$$\Theta = (|V_G^-|^2 + |V_L^-|^2)/(|V_G^+|^2 + |V_L^+|^2). \tag{24}$$

Note that in the case of a single-port scattering set-up discussed earlier in this section, the Θ-function collapses to the gain/loss side reflectances. Let V_L^+/V_G^+ be a generic ratio, then the perfect amplifier coefficient [1] is obtained as:

$$\Theta_{amp}(\omega) = \frac{\left|\frac{V_L^+}{V_G^+}\mathcal{M}_{12}(\omega) + 1\right|^2 + \left|\frac{V_L^+}{V_G^+} - \mathcal{M}_{21}(\omega)\right|^2}{\left(1 + \frac{|V_L^+|^2}{|V_G^+|^2}\right)\left|\mathcal{M}_{22}(\omega)\right|^2}. \tag{25}$$

At the singularity frequency point $\omega = \omega_J$, the $\Theta(\omega)$-function diverges as $\omega \to \omega_J$ and the circuit acts as an amplifier/laser. If on the other hand, we assume that $V_L^+ = \mathcal{M}_{21}(\omega)V_G^+$ (perfect adsorption condition), we can obtain [1]

$$\Theta_{abs}(\omega_J) = (|\mathcal{M}_{22}(\omega_J)\mathcal{M}_{11}(\omega_J)|^2)/(1 + |\mathcal{M}_{21}(\omega_J)|^2|\mathcal{M}_{11}(\omega_J)|^2) = 0. \tag{26}$$

3 Measurement Theory of Scattering Properties

3.1 Measurement Theory of Single-port Scattering

In Section 2, we derived the theoretical formula of single-port scattering coefficient. However, the theoretical formula cannot be used to calculate the coefficients for simulation and measurement.

Therefore, we need to find feasible method for practical measurement. We take the Supplementary Figure 6 for example. It is obvious to know that $V_G = V_G^+ + V_G^-$. Here, V_G is the node voltage of gain side; V_G^+ is the incident wave, and V_G^- is the reflected wave. We rewrite the reflection of gain side r_G in Eq. (10) as

$$r_G = \frac{\mathcal{R}_G(\omega) - Z_0}{\mathcal{R}_G(\omega) + Z_0} = \frac{V_G^-}{V_G^+} = \beta_G e^{j\phi}, \tag{27}$$

where, β is the module of reflection coefficient, and ϕ is the phase of reflection coefficient. Therefore, $V_G = V_G^+ + V_G^- = V_G^+ \cdot (1 + \beta_G e^{j\phi}) = V_G^+ \cdot (1 + \beta_G \cos(\phi) + \beta_G \sin(\phi))$, and

$$\frac{V_G}{V_G^+} = 1 + \beta_G \cos(\phi) + \beta_G \sin(\phi). \tag{28}$$

Here, $V_G^+ = V_1/2$. V_1 is the voltage of V_{S1}.

On the other hand, we also can let

$$\frac{V_G}{V_G^+} = \alpha e^{j\xi} = \alpha \cos(\xi) + j\alpha \sin(\xi). \tag{29}$$

Here, V_G is the node voltage of gain side; ξ is the phase difference between V_G and source voltage V_{S1}; α is the amplitude ratio between V_G and incident wave voltage V_G^+. Comparing Eq. (28) and Eq. (29), we can obtain

$$\begin{cases} \beta_G \sin(\phi) = \alpha \sin(\xi); \\ 1 + \beta_G \cos(\phi) = \alpha \cos(\xi). \end{cases} \tag{30}$$

Then

$$\begin{cases} \beta_G = \sqrt{1 + \alpha^2 - 2\alpha \cos(\xi)}; \\ \phi = \arctan((\alpha \sin(\xi))/(\beta_G \cos(\xi) - 1)). \end{cases} \tag{31}$$

Therefore, if we measure the amplitude of V_G and the phase difference ξ between V_G and source voltage V_{S1}, we can get the experiment results of reflection coefficient r_G of gain side. Similarly, r_L of loss side can also be obtained.

3.2 Measurement Theory of Two-port Scattering

From Supplementary Figure 8 and Eq. (27), we can easily get

$$\begin{cases} V_G^- = V_G^+ \cdot \beta_G e^{j\phi_1}; \\ V_L^- = V_L^+ \cdot \beta_L e^{j\phi_2}. \end{cases} \tag{32}$$

Then, the formula for measurement is

$$\Theta = \frac{|V_G^-|^2 + |V_L^-|^2}{|V_G^+|^2 + |V_L^+|^2} = \frac{|V_L^+ \cdot \beta_L e^{j\phi_2}|^2 + |V_G^+ \cdot \beta_G e^{j\phi_1}|^2}{|V_G^+|^2 + |V_L^+|^2}. \tag{33}$$

Here, $V_G^+ = V_1/2$, and $V_L^+ = V_2/2$. V_1 and V_2 are the amplitude of V_{S1} and V_{S2}, respectively. Base on the measurement theory of Section 3.1, ϕ_1, ϕ_2, β_L and β_G could be easily calculated. Therefore, Θ can be obtained by plugging these values into Eq. (33).

4 Microwave Generation

4.1 Frequency Tuning Range Comparison

Microwave generation is very important on diverse on-chip applications [11, 12, 13, 14, 15, 16]. We theoretically compare the bandwidth of microwave generation of the fully integrated PT-symmetric electronic system and traditional oscillators. As derived in the Methods of the main text, the fully integrated PT-symmetric electronic system (Supplementary Figure 9a) has four normal mode frequencies,

$$\omega_{1,2} = \pm\frac{\sqrt{\gamma_{EP}^2 - \gamma^2} + \sqrt{\gamma_{UP}^2 - \gamma^2}}{2\sqrt{1+2c}}, \quad \omega_{3,4} = \pm\frac{\sqrt{\gamma_{EP}^2 - \gamma^2} - \sqrt{\gamma_{UP}^2 - \gamma^2}}{2\sqrt{1+2c}}, \tag{34}$$

where, the breaking point (γ_{EP}) and the upper critical point (γ_{UP}) are identified as

$$\gamma_{EP} = \left|1 - \sqrt{1+2c}\right|, \quad \gamma_{UP} = 1 + \sqrt{1+2c}. \tag{35}$$

The corresponding phase difference [1] between the two RLC resonators can be expressed as

$$\phi_{1,3} = \frac{\pi}{2} - \tan^{-1}\left[\frac{1}{\gamma} \cdot (\frac{1}{\omega_{1,3}} - (1+c) \cdot \omega_{1,3})\right]. \tag{36}$$

Here, γ is the gain-loss contrast tuning which is defined as $\gamma = \sqrt{L/C}/R$.

We then derive the theory for conventional single-core oscillators (Supplementary Figure 9b). Applying Kirchoff's law on the equivalent circuit representation in Supplementary Figure 9d yields the following expression:

$$\frac{V_{GP}}{-R/2} + \frac{V_{GP}}{i\omega' L/2} + V_{GP} \cdot i\omega' 2C = 0. \tag{37}$$

Here, $R = -R_G || R_0$ with $-R_G$ the tunable gain and R_0 the inherent loss of the resonator. Using the same normalization methods presented before, that is $\omega_0 = 1/\sqrt{LC}$, $\gamma = \sqrt{L/C}/R$, Eq. (37)

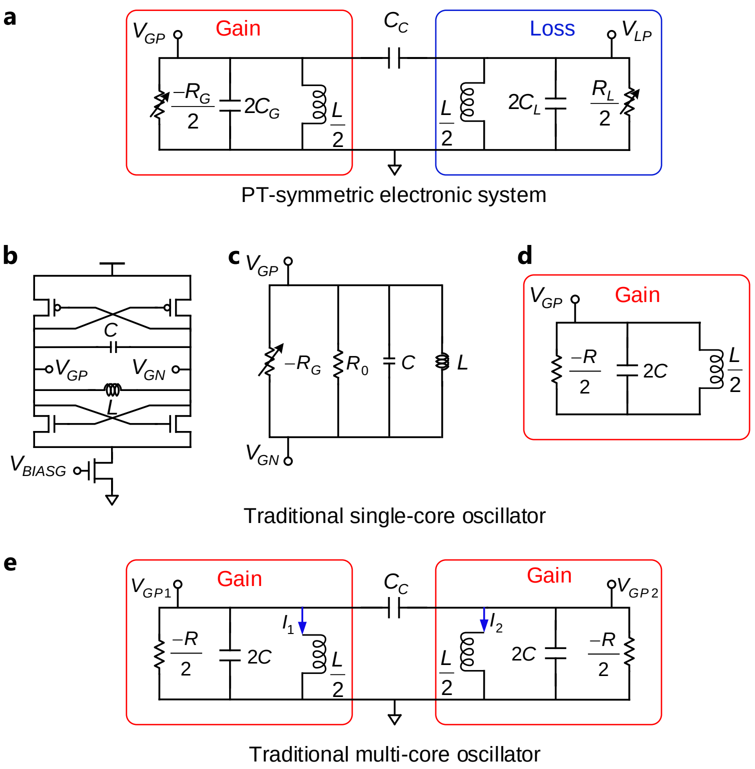

Supplementary Figure 9: Illustration of three kinds of microwave generators. **a**. The equivalent single-ended circuit schematic of the differential architecture of the fully integrated PT-symmetric electronic system. This figure is taken from Supplementary Figure 3b. **b**. Schematic view of a traditional single-core oscillator. **c**. Equivalent differential model of the single-core oscillator. **d**. The equivalent single-ended circuit schematic of the single-core oscillator. **e**. The equivalent single-ended circuit schematic of multi-core oscillators. We use the multi-core oscillator built upon two capacitively coupled active RLC resonators as an example.

can be transferred into $\omega^2 + i\gamma\omega - 1 = 0$ whose solutions are

$$\omega_{1,2} = \frac{-i\gamma \pm \sqrt{4-\gamma^2}}{2}. \tag{38}$$

Eq. (38) suggests that the oscillation happening in a single-core oscillator mainly goes through two phases: start-up phase and stable phase. In the start-up phase, a small-signal gain $-R_G$ initially set slightly above the inherent loss R_0 is used to compensate for the loss so as to generate an oscillated microwave. The oscillation frequency–the real part of the microwave–is in fact related to the amount of loss. However, as the amplitude of the microwave exponentially grows, the small-signal gain $-R_G$ is degenerated in the large-signal domain due to the nonlinearity of the system, whose final value is equivalent to the loss R_0, leading to $\gamma \to 0$. The oscillation then steps into the stable phase, where the microwave's amplitude saturates at a fixed amplitude level and its oscillation frequency also becomes stable. Such an oscillation frequency is independent of the gain-loss contrast and only

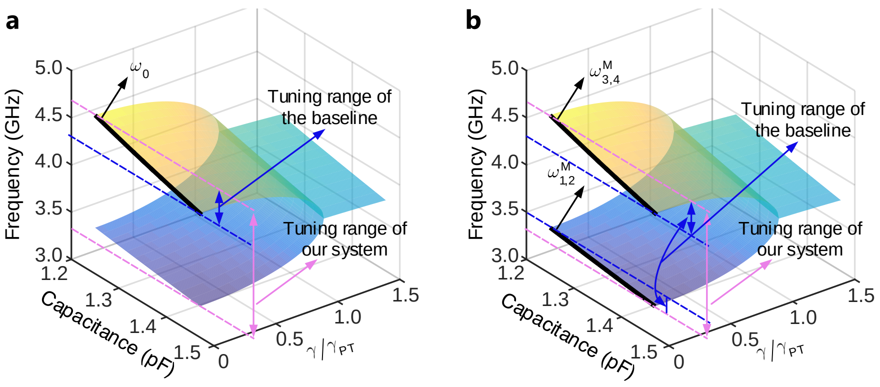

Supplementary Figure 10: Theoretical comparisons of frequency tuning range between different oscillators. **a**. Comparison between the single-core oscillator and our system. The black line indicates the tuning range of the single-core oscillator. **b**. Comparison between the multi-core oscillator and our system. The black line indicates the tuning range of the multi-core oscillator.

determined by the natural frequency ($\omega_0 = 1/\sqrt{LC}$) of the resonator, i.e.,

$$\omega_{1,2} = 1. \tag{39}$$

Note that in this stable phase, an oscillator generates stable sinusoidal waves for diverse on-chip applications. Obviously, the stable oscillation frequency of conventional single-core oscillators can be tuned only by the capacitance C or the inductance L.

Multi-core VCOs are formed by coupling multiple identical single-core LC VCOs. Here, we use a multi-core VCO built upon two coupled resonators as shown in Supplementary Figure 9e as an example. This oscillator with coupled-resonator structure without gain-loss contrast is used as another baseline of our system. A similar I-V relations of the circuit can be obtained by using the Kirchoff's law:

$$\begin{cases} V_{GP1} = i\omega' \dfrac{L}{2} \cdot I_1, \quad I_1 - \dfrac{V_{GP1}}{R/2} + i\omega' 2C \cdot V_{GP1} + i\omega' C_C \cdot (V_{GP1} - V_{GP2}) = 0; \\ \\ V_{GP2} = i\omega' \dfrac{L}{2} \cdot I_2, \quad I_2 - \dfrac{V_{GP2}}{R/2} + i\omega' 2C \cdot V_{GP2} + i\omega' C_C \cdot (V_{GP2} - V_{GP1}) = 0. \end{cases} \tag{40}$$

Using the same normalization methods as the single-core VCOs and considering $\gamma \to 0$, the solutions are given by

$$\omega^{\mathrm{M}}_{1,2} = \pm \frac{1}{\sqrt{(1+2c)}}; \quad \omega^{\mathrm{M}}_{3,4} = \pm 1. \tag{41}$$

Comparing Eq. (34), Eq. (39), and Eq. (41), it can be found that Eq. (39) is a special form of $\omega_{1,2}$ in Eq. (34) when $\gamma \to 0$; Eq. (41) is a special form of Eq. (34) when $\gamma \to 0$. The comparison shows that in addition to the inherent tuning freedoms preserved by ω_0, $\omega_{1,2}$ and $\omega_{3,4}$ in Eq. (34) also

preserve an extra resistive tuning freedom, i.e., $\gamma = \sqrt{L/C}/R$. Supplementary Figure 10 compares the theoretical frequency tuning range of three oscillators using the same tunable parameters, exhibiting a larger tuning range of our system.

4.2 Phase Noise Comparison

Supplementary Figure 11a shows the passive resonator model for a conventional single-core oscillator whose phase noise (PN) model is illustrated in Supplementary Figure 11b. The main noise sources come from resistor thermal noise ($I_{R_0}^2(\omega) = 4kT/R_0,\ \omega > 0$) and transistor thermal noise ($I_{g_m}^2(f) = 4kTmg_m,\ \omega > 0$). The classical PN formula of the conventional single-core oscillators [17] is shown below

$$\begin{aligned}\mathcal{L}_{conv}(\triangle\omega) &= 10 \cdot \log\left[\frac{\mathcal{P}_{sideband}(\omega + \triangle\omega, 1Hz)}{\mathcal{P}_{carrier}}\right] \\ &= 10 \cdot \log\left((1+m) \cdot \frac{4kTR_0}{V_{osc,conv}^2} \cdot \left(\frac{\omega}{2Q_S\triangle\omega}\right)^2\right).\end{aligned} \tag{42}$$

Here, $\mathcal{P}_{sideband}(\omega + \triangle\omega, 1\ Hz)$ represents the single sideband power of noise at a frequency offset of $\triangle\omega$ from the carrier with a measurement bandwidth of 1 Hz. ω is the oscillation frequency. $\triangle\omega$ is the frequency offset. k is Boltzmann's constant. T is the absolute temperature. R_0 is the inherent resonator resistance. m is a noise factor of the active device. $V_{osc,conv}$ is the oscillation amplitude. Q_S is the quality factor of the resonator as shown in Supplementary Figure 11a, defined as $Q_S = \omega R_0 C = R_0/(\omega L)$.

It is well-known in the oscillator filed that multi-core oscillators built upon N identically coupled resonators can lead to the PN reduction by $10\log_{10} N$ dB as compared to a single-core oscillator [18, 19, 20, 21]. A detailed theoretical analysis is proposed in a previous work [18]. We provide an intuitive understanding here by using a multi-core oscillator composed of two coupled resonators as an example. Supplementary Figure 11c shows the passive resonator model for the multi-core oscillator. One can imagine that the two coupled resonators can be equivalently considered as a single-core resonator with doubled capacitance, halved inductance and halved inherent resistor as shown in Supplementary Figure 11d. Then, the oscillation frequency remains the same as the single-core oscillator. According to Eq. (42), PN is reduced by 3 dB in this case. This case study indicates that although the noise sources of two coupled resonators double, the effective Q-factor (Q_C) of the system (Figure 11e) also doubles, i.e.,

$$\mathcal{L}_c(\triangle\omega) = \mathcal{L}_{conv}(\triangle\omega) - 3 = 10 \cdot \log_{10}\left((1+m) \cdot \frac{8kTR}{V_{osc,conv}^2} \cdot \left(\frac{\omega}{2 \cdot 2Q_S\triangle\omega}\right)^2\right), \tag{43}$$

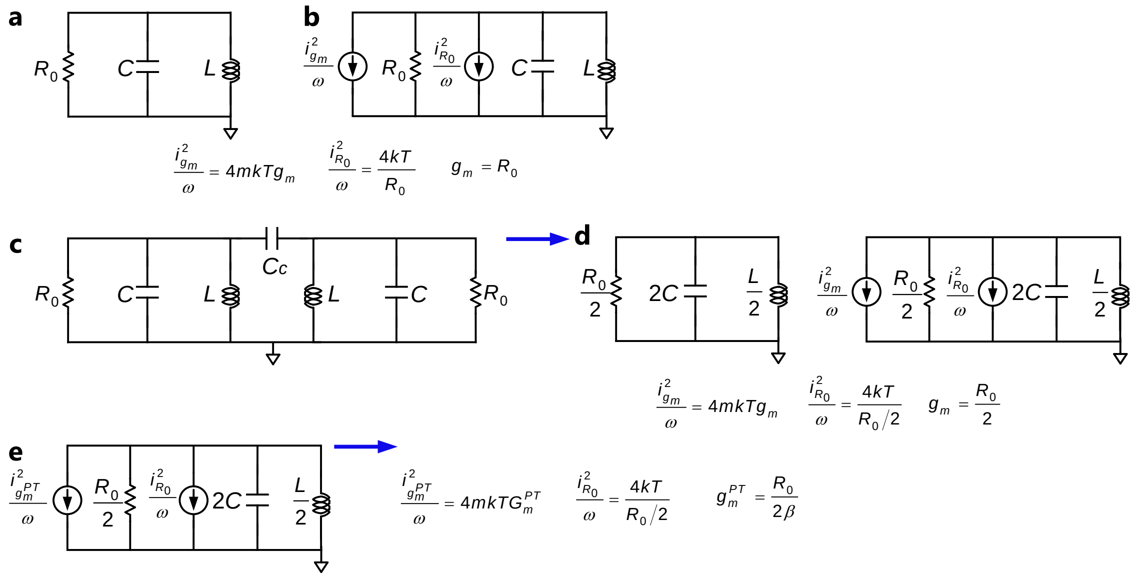

Supplementary Figure 11: Phase noise models of different oscillators. **a**. Passive resonator model of a conventional single-core oscillator. **b**. Phase noise model of the single-core oscillator. **c**. Passive resonator model of a conventional multi-core oscillator based on two coupled resonators. **d**. Equivalent resonator model and phase noise model for the multi-core oscillator. **e**. Equivalent phase noise model of our system.

where $Q_C = 2Q_S$. Our system built upon two coupled resonators also obey this rule. However, with the unique gain-loss contrast tuning, our system achieves more PN reduction. In conventional oscillators, the provided gain only demands to cancel the inherent loss. However, in our system, the provided gain not only needs to compensate for the inherent loss, but also requires to balance the tunable loss. Assuming the ratio between the provided gain and the inherent loss is β ($\beta > 1$), the PN of our system is expressed as

$$\mathcal{L}_{PT}(\triangle\omega) = 10 \cdot \log_{10}\left((1+\beta m) \cdot \frac{8kTR}{(\beta^2 V_{osc,conv})^2} \cdot \left(\frac{\omega}{2 \cdot 2Q_S \triangle\omega}\right)^2\right). \tag{44}$$

Here, the oscillation amplitude of our system increases to $\beta^2\times$ as the current flowing into the resonator is quadratically proportional to the gain. Note that in the saturation region, the resonator current I_D is linear with the square of transconductance g_m^{PT} based on the I-V relationship of MOSFET: $g_m^{\mathrm{PT}} = \partial i_D/\partial V_{GS} = \mu_n C_{ox}(W/L)(V_{GS} - V_{th}) = \sqrt{2\mu_n C_{ox}(W/L)I_D}$. Comparing Eq. (42) and Eq. (44), we obtain

$$\mathcal{L}_{PT}(\triangle\omega) = \left[\mathcal{L}_c(\triangle\omega) - 10\log_{10}\left(\frac{\beta^4(1+m)}{1+\beta m}\right) - 3\right] < \mathcal{L}_c(\triangle\omega) - 3. \tag{45}$$

Eq. (45) shows that the gain-loss contrast tuning of our system can further reduce PN by increasing the power of carrier. Therefore, the PN improvement of our system is attributed to two facts: 1)

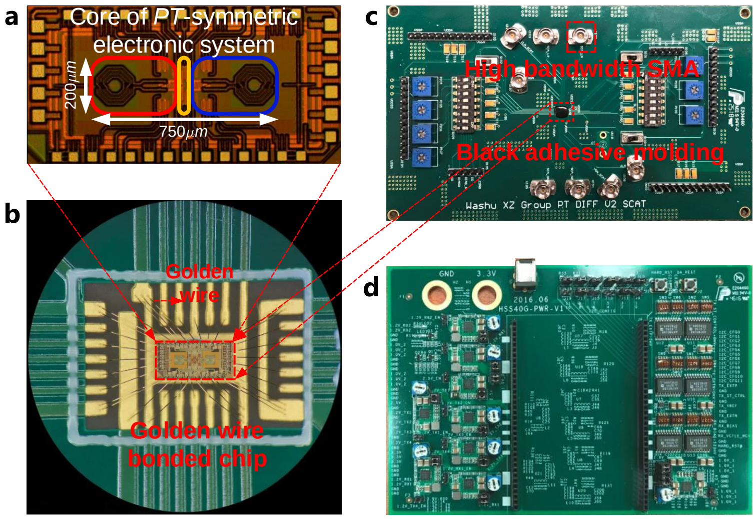

Supplementary Figure 12: Experimental setups. **a**. Chip die photo. **b**. Bonding diagram. **c**. Daughter PCB for controlling the biases of the IC components. **d**. Mother board for power supply.

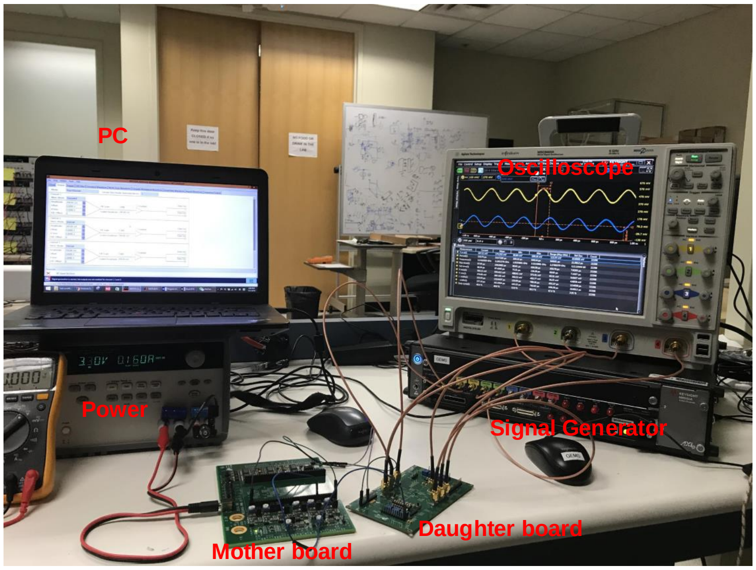

Supplementary Figure 13: Complete setup to test the system. The test platform consists of a daughter board, a mother board, a power, a signal generator, an oscilloscope, and a PC.

the coupled-resonator structure of our system can enhance the effective Q-factor of the system, and 2) the gain-loss contrast tuning can increase the oscillation amplitude, decreasing the effect of noise.

5 Experiments

5.1 Experimental Setup

We fabricated the chip with a 130 nm CMOS technology. The chip die photo is shown in Supplementary Figure 12a. The core area of the system is $200\mu m \times 750\mu m$. To test the chip, we

designed two printed circuit boards (PCBs). One is a daughter board (Supplementary Figure 12c) and the other one is a mother board (Supplementary Figure 12d). The system chip was bonded on the daughter board by gold wires (Supplementary Figure 12b). The daughter board provides all the control signals and high-speed inputs (outputs) for the chip, such as gain (loss) bias voltage, varactor bias voltage. All the high-speed input/output terminals of the chip are accessed by high bandwidth surface mount ahead (SMA) on the daughter board. The mother board is used as power supply for the daughter board. A complete setup is shown in Supplementary Figure 13. Our experimental setup consists of a bonded chip in a daughterboard, a motherboard, a power supply, a mixed signal oscilloscope (MSO, Agilent 9404A), an arbitrary wave generator (AWG, KEYSIGHT M8195A) and a personal computer (PC). The MSO has four pairs of differential channels, and its highest sampling rate is 20 GSa/s. The AWG has four pairs of differential channels, each pair of which can generate arbitrary waves up to 50 GHz with independently varying phase.

5.2 Experiment And Simulation Procedures

In the phase transition experiments, the outputs of two RLC resonators were connected to the MSO. To test the PT-symmetry spontaneous breaking, we used the zig-zagging method to make either eigen-frequency dominant [1, 22].

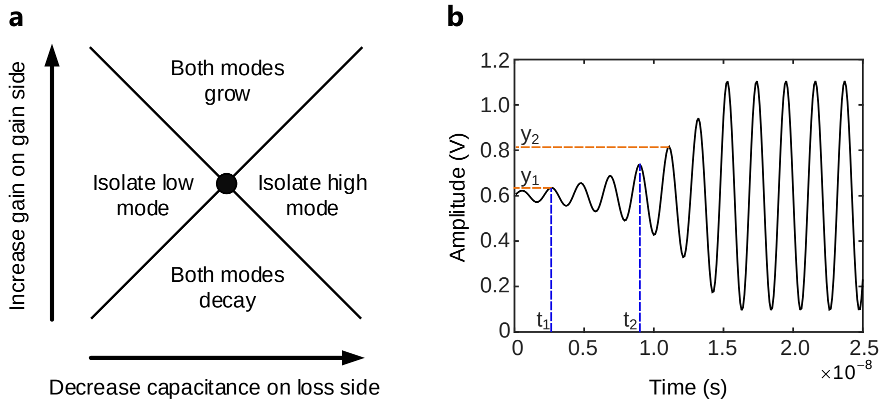

Supplementary Figure 14: Measurement methodology to obtain the eigenfrequencies of our system [22]. **a**. The 'X' plane used as the instructions to manually tune our system. **b**. A simplified example to show how to obtain the imaginary part of eigenfrequencies.

The method is originally proposed in [22], which can be best introduced using Supplementary Figure 14**a**. The horizontal axis represents the capacitance difference $\triangle C = C_L - C_G$ between the two resonators. "Moving rightward on the figure indicates decreasing $\triangle C$. The vertical axis is the gain. Upward movements indicate increasing gain. The dot at the center of the X represents a point where the gain and loss are exactly balanced, but the capacitance is imbalanced by a small amount $\triangle C > 0$. The goal is to attain a stable dimer configuration just barely below that center

dot. Then the slightest changes can cause a marginal instability in one, the other, or both modes, allowing each mode to be observed individually in a state of gain-loss balance. An algorithm to find this balance point by zig-zagging along one of the bottom boundary lines of the X follows: 1) Reduce gain until all modes decay by ending just inside a border of the X; 2) Change $\triangle C$ a bit in whichever direction doesn't immediately cause instability; 3) Increase gain until something oscillates; 4) Change $\triangle C$ enough to kill the oscillation, then a bit further; 5) Repeat 3) and 4) until tiny capacitance changes cause a switching from the high frequency to low frequency zone, with only a tiny "dead zone" in between. Note that as $\gamma \to \gamma_{EP}$, $\triangle C \to 0$. Beyond γ_{EP}, $\triangle C$ was held fixed at its asymptotic value."

In the exact phase, mode frequencies were directly observed by balancing gain-loss and slightly unbalancing the capacitance, then correcting for the imbalance. For each mode, once the system was brought to a state of marginal oscillation, oscilloscope waveform capture recorded $V_G(t)$ and $V_L(t)$, the voltage data at each side of the system. These data were analyzed for real frequency and amplitude. This process described above forced the imaginary part of the frequency to be zero, and so the imaginary frequency data was automatically recorded as zero. In the broken phase, the capacitance trim is kept fixed at its asymptotic value, and the gain trim is set to a bit higher than center dot. The exponential growth of transient data obtained in Figure 14**b** then directly gives us the imaginary component: $\omega_{\text{Im}} = (\ln(y_2 - V_{\text{cm}}) - \ln(y_1 - V_{\text{cm}}))/(t_2 - t_1)$. Here, V_{cm} is the common mode voltage, which is set to be $V_{\text{DD}}/2 = 0.6V$ in our design. Note that only a piece of the transient curve as shown in Figure 14**b** is used to calculate the imaginary part. As the amplitude increases, the gain will be degenerated due to the nonlinearity of the system, which can lead to the computation errors.

In the single-port scattering experiments, the system was biased in the exact phase. Then, a sinusoidal signal with varied frequency was applied into the system. Note that the signal power was chosen to set the system in the linear region. The incident wave V_G^+ (V_L^+) and the reflected wave V_G^- (V_L^-) were extracted from the voltages at either side of the TL, from which the scattering coefficients $r_G = V_G^-/V_G^+$ and $r_L = V_L^-/V_L^+$ were calculated. In the two-port scattering simulations, the AWG sourced sinusoidal signals with varying frequencies or phase into the chip through TL. Then signals on both terminals of the TL were sent into the MSO such that the incident wave and reflected wave could be captured. Theoretically, the ideal case of $\Theta_{abs} = 0$ and $\Theta_{amp} = \infty$ can only occur when the gain and loss are perfectly balanced. In our system, small imbalance of RLC components existed in the two RLC circuits due to minor fabrication error, which could not be completely compensated by the external tuning. Such a tiny imbalance resulted in a large

deviation of theoretical Θ_{abs} and Θ_{amp} of our system from the ideally balanced condition (see Supplementary Figure 17a), and experimental difficulty in measuring Θ_{abs}. Therefore, we performed SPICE simulation with special scanning techniques [22] to obtain the corresponding results with $\Theta < 0$ in Supplementary Figure 17b. When the PT-symmetric dimmer acts as a perfect absorber, the condition $V_L^+ = \mathcal{M}_{21}(\omega)V_G^+$ must be satisfied. In the simulation, we let $V_L^+ = Ae^{i\phi'}(\omega)V_G^+$. Each lower data point near the absorption point in Supplementary Figure 17b was found by fixing frequency and scanning through values of $\phi' = 90°$ in tightly spaced increments, then recording the minimum Θ value. Within these ϕ' scans, an iterative process of measurement and resetting was used at each step, to ensure that A and ϕ' were within a small tolerance level of the theoretically specified values. The portion of the bottom (blue) curve in Supplementary Figure 17b near the minimum is an example of one of these high-precision scans.

In the nonreciprocal experiments, the AWG fed sinusoidal signals with varying frequencies into the system through the gain (loss) side TL. Then both the incident wave on the input terminal of the gain (loss) side TL and the reflected wave on the output terminal of loss (gain) side TL could be captured by MSO.

6 Supplementary Results

6.1 Comparisons of Microwave Generation

In our system, a conventional oscillator (Supplementary Figure 9b) can be obtained by turning off SW1 to decouple the two RLC resonators in the fully integrated PT-symmetric electronic system shown in Supplementary Figure 1. We compare the phase noise performance of the baseline oscillator and our PT-symmetric system at different frequencies (low, medium and high frequency in each individual system) in Supplementary Figure 15. The experimental results show that the fully integrated PT-symmetric electronic system generally has better phase noise performance in the tuning range than the conventional oscillator. Table 1 summarizes the comparison.

We then further examine the Eq. (34) and Eq. (36) in Section 4.1 at the coalescence frequency $\omega_1 = \omega_3$. We find that $\phi_{1,3} = \pi/2$ if $\omega_1 = \omega_3 = 1/\sqrt{(1+c)}$. This indicates that by carefully choosing design parameters at EP, the phase difference between two sides is $\pi/2$, then we can achieve quadrature microwave generation [23, 24, 25, 26]. Note that we use a differential architecture to design the system, therefore the phase for V_{GP}, V_{GN}, V_{LP}, V_{LN} is 0, π, $\pi/2$, $3\pi/2$. Supplementary Figure 16 shows the experimental results of the quadrature microwave generation enabled by our system.

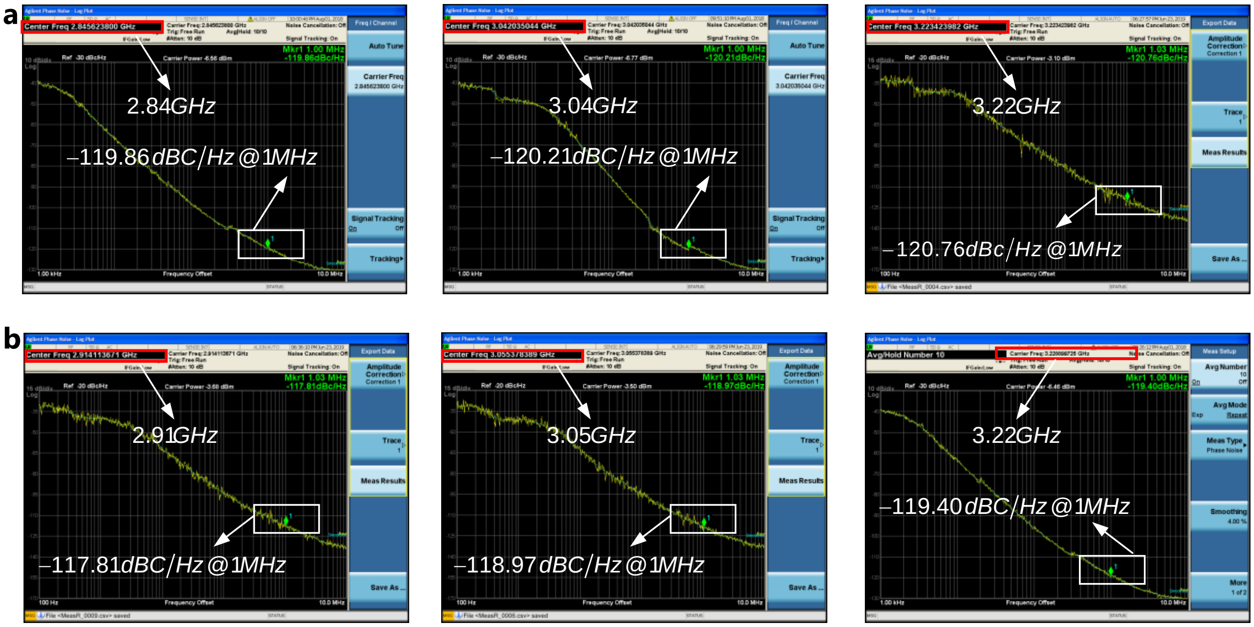

Supplementary Figure 15: Comparison of phase noise performance of the microwave generation between the fully integrated PT-symmetric electronic system and the baseline conventional oscillator with a single-resonator structure. **a**. Phase noise of our system at different frequencies: 2.84 GHz (low frequency), 3.04 GHz (medium frequency), 3.22 GHz (high frequency). **b**. Phase noise of the baseline oscillator at different frequencies: 2.91 GHz (low frequency), 3.05 GHz (medium frequency), 3.22 GHz (high frequency).

Supplementary Table 1: Comparisons of microwave generation between the fully integrated PT-symmetric electronic system and the baseline traditional oscillator.

Works	Baseline Oscillator	Our PT-symmetric System
Technology (nm)	130	130
Supply (V)	1.2	1.2
Power (mW)	4.31	4.31
Area (mm^2)	0.15	0.15
f_{min} (GHz)	2.93	2.63
f_{max} (GHz)	3.23	3.20
LC Tuning parameter	$L = 1.85$ nH; $C \in [1.35, 1.55]$ pF	$L = 1.85$ nH; $C_C = 500$ fF; $C \in [1.35, 1.55]$ pF.
R tuning	N/A	$R \in [80, 260]$ Ω
FTR (%)	9.70	20.17
PN(dBc/Hz@1MHz) (Average)	118.72	120.28

6.2 Scattering Results

Theoretically, the ω_J is uniquely determined by the tuning parameter $\gamma = \sqrt{(L/C)}/R$ when the system is perfectly balanced. A small variation of gain/loss value R in γ will cause the significant deviation of Θ_{amp} and Θ_{abs} from ideal value ($\Theta_{amp} = \infty$, $\Theta_{abs} = 0$). Supplementary Figure 17a shows the theoretical simulation of several groups Θ_{amp} and Θ_{abs} under different variation of R. $R = 100$ Ω can be consider as the case to achieve ideal Θ_{amp} and Θ_{abs}. Even if there is only 15 Ω deviation, the deviation of Θ_{amp} and Θ_{abs} is up to 80 dB. Considering the fabrication process leads

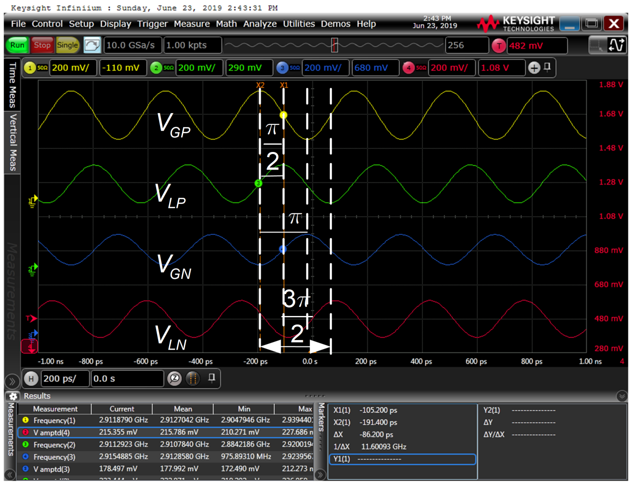

Supplementary Figure 16: Example of quadrature microwave generation enabled by the PT-symmetric electronic system. The system is biased around the exceptional point.

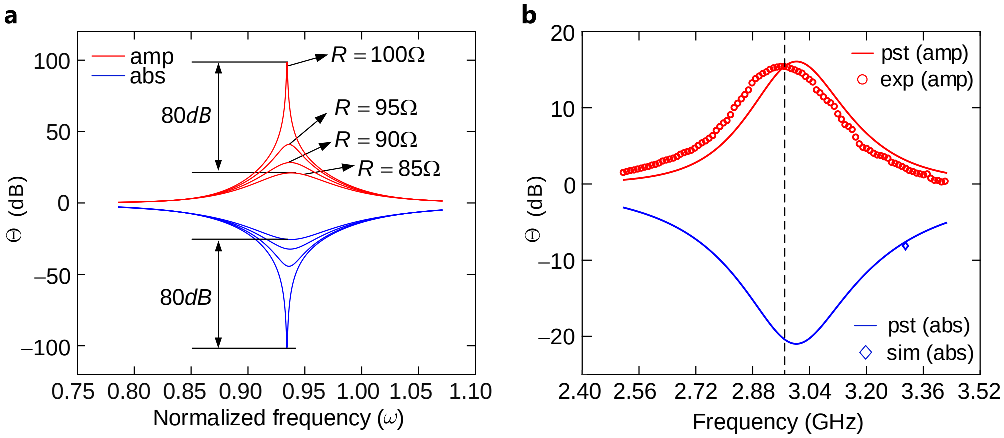

Supplementary Figure 17: Simulations and experiments of two-port scattering property. a. Theoretical deviation of Θ_{amp} and Θ_{abs} under different gain (loss) value R. **b.** Measured results.

to the imbalance of CMOS components between the two RLC resonators which cannot be completely compensated by the external tuning, the practical deviation becomes worse. Supplementary Figure 17b demonstrates a measured result (red dots) of two-port scattering property when the system suffers from small fabrication mismatches.

6.3 Non-reciprocal Microwave Transmissions

Extra experimental results of non-reciprocal transmission are shown in Supplementary Figure 18 which together with the Figure 4a-e (main text) show the non-reciprocal trend of the isolation in Figure 4f (main text). Our systems shows strong isolation among a wide bandwidth in the microwave domain. Our system also requires lower power threshold and show interesting insertion gain due to enhanced nonlinearity enabled by PT-symmetry as compared with traditional nonlinearity-induced

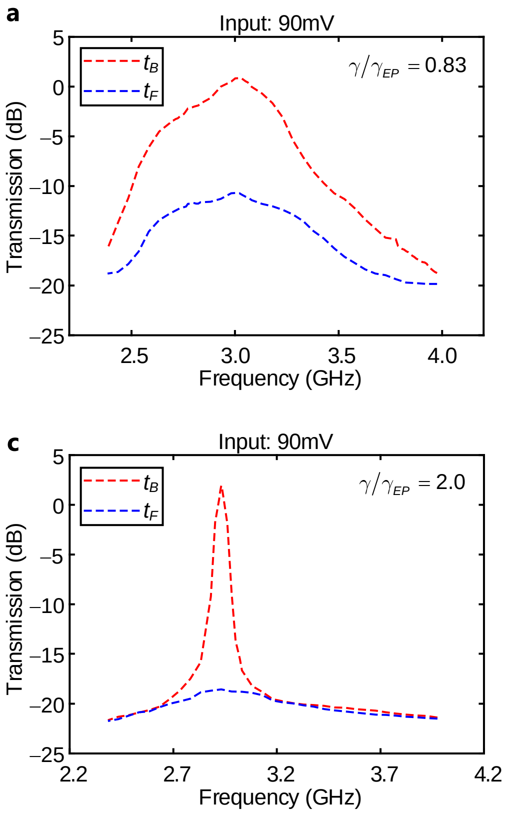

Supplementary Figure 18: Supplementary experimental results for non-reciprocal transmission. a. Nonreciprocal transmission is observed in the exact phase ($\gamma/\gamma_{EP} = 0.83$) with two peaks, where the forward transmission is up to -10.5 dB while the backward transmission is 1.0 dB. **b**. Nonreciprocal transmission is observed in the exact phase ($\gamma/\gamma_{EP} = 0.9$) with two peaks, where the forward transmission is up to -14.5 dB while the backward transmission is 1.9 dB. **c**. Nonreciprocal transmission is observed in the broken phase ($\gamma/\gamma_{EP} = 2$) with one peak, where the forward transmission is up to -18.7 dB while the backward transmission is 2.1 dB.

Supplementary Table 2: Non-reciprocity comparisons between our system and state-of-the-art isolators based on nonlinearity.

Works	Our System	PRL '13 [27]	Nature Electron '19 [28]	Nature Electron '20 [29]
Power threshold	−21 dBm	9 dBm	17 dBm	−20 dBm
Isolation	20 dB	<5 dB	35 dB	10 dB
Bandwidth	[2.75 ∼ 3.10] GHz	[38 ∼ 40] KHz	[700 ∼ 800] MHz	200 MHz
Insertion gain?	5 dB	No	No	No
Fully integrated?	Yes	No	No	No

isolation (see Supplementary Table 2).

7 Versatile Fully Integrated PT-symmetric Electronic System

In addition to the proposed architecture based on the gain (loss) tuning, it is straightforward for us to implement other architectures for the system by leveraging the flexible tuning mechanisms of IC. We propose two variants of the system by using 130 nm CMOS technology and show them

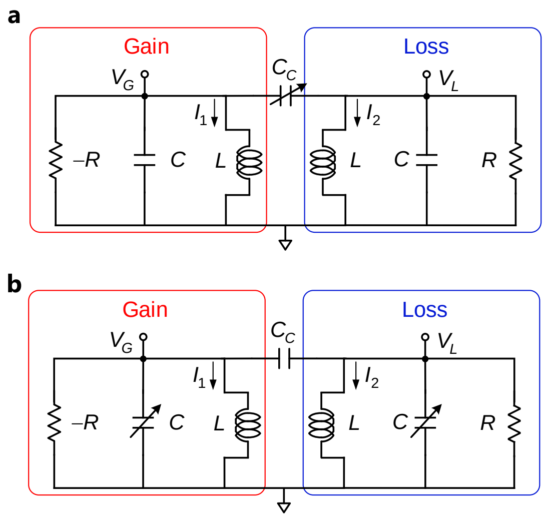

Supplementary Figure 19: Versatile architectures to implement fully integrated PT-symmetric electronic system. a. Coupling-tuning architecture. In this architecture, only coupling capacitance C_C is adjustable, all other components are fixed. b. Capacitive tuning architecture. In this architecture, only capacitance in the RLC resonators is adjustable, all other components are fixed.

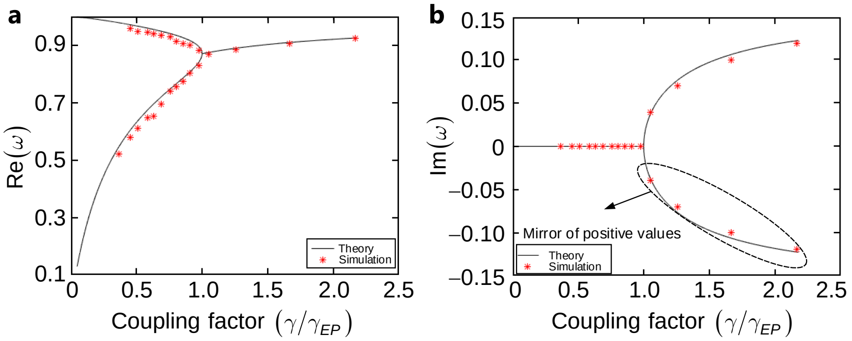

Supplementary Figure 20: Phase transition of the system based on coupling-tuning architecture. a. Real part of the eigenfrequencies. **b**. Imaginary part of the eigenfrequencies. The part below the zero axis is the symmetrical part of the positive one. Star symbols are simulations while lines are theoretical prediction.

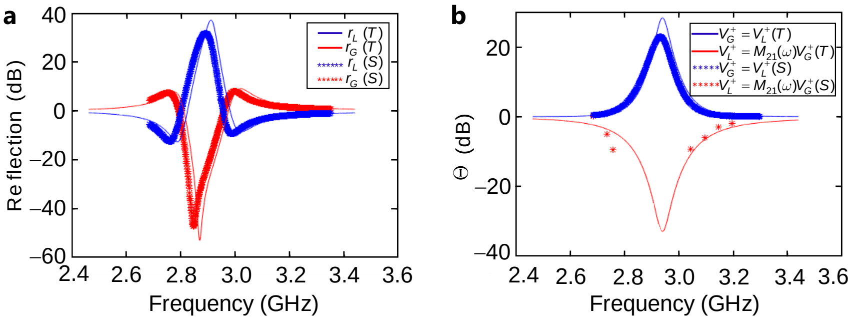

Supplementary Figure 21: Scattering properties of the system based on coupling-tuning architecture. a. Single-port scattering. **b**. Two-port scattering [1]. Star symbols are simulation (S) results while lines are theoretical (T) prediction.

in Supplementary Figure 19. The first variant is based on coupling-tuning architecture (Supplementary Figure 19a). In this architecture, all the components are fixed except for the coupling

capacitance C_C. The C_C can be realized by switched-capacitor arrays or varactors. The second variant uses capacitance-tuning mechanism (Supplementary Figure 19b). In this architecture, all the components are fixed except for the RLC resonator's capacitance C_G (C_L). The C_G (C_L) can also be realized in the same way as C_C. Although they are different in tuning mechanisms, the theory of the PT-symmetry spontaneous breaking keeps the same. Based on Eq. (35), in the coupling-tuning architecture, γ_{EP} and γ_{UP} are evolving with the capacitance ratio c while γ is fixed. In the capacitance-tuning architecture, all γ, γ_{EP} and γ_{UP} are evolving with the RLC resonator's capacitance C, but γ_{EP} and γ_{UP} change faster.

We simulate the first variant of the system by using 130 nm CMOS technology and show the corresponding results in Supplementary Figure 20 and Figure 21. The design parameters of this variant are $L = 3.50$ nH, $C_C \in [0.3, 1.3]$ pF, $C = 0.4$ pF, and $R = 270\ \Omega$. All the simulation results are matched with theoretical predictions, demonstrating that ICs can provide versatile structures to study PT-symmetric electronics.

8 Extended Discussions

8.1 Discussion on Periodic PT-symmetric Electronic Structures

PT-symmetric periodic structures, near the spontaneous PT symmetry breaking point, can act as unidirectional invisible media. In this regime, the reflection from one end is diminished while it is enhanced from the other. In electronics, the unidirectional invisibility has been studied by using diverse board-level PT-symmetric systems [30, 31, 32].

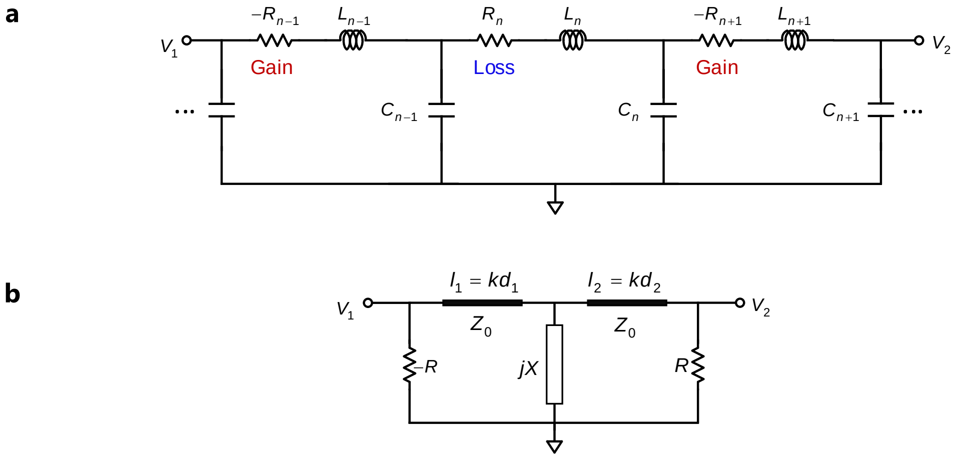

Supplementary Figure 22: Circuit structure for unidirectional invisibility based on PT-symmetric electronic circuits. **a**. Circuit structure for unidirectional invisibility based on a periodic PT-symmetric transmission line circuit [32]. **b**. The circuit schematic used in the previous work [30].

Supplementary Figure 22a shows a circuit schematic of a PT-symmetric periodic structure based

on a transmission line model [32], where the resistor R_n is distributed according to the configuration of PT symmetry composed of a gain ($-R$) and loss ($+R$) sequence. Theoretical analysis suggests that such a structure shows PT symmetry phase transition from real to complex eigenvalues as a function of resistance R. It can be used as a counterpart of PT-symmetric Bragg periodic structures in the electronic domain to study the unidirectional invisibility around the exceptional point.

The system in reference [30] is composed of lumped elements and transmission lines as shown in Supplementary Figure 22b. The two parallel resistors are separated by two transmission lines of which the electric lengths are $l_1 = kd_1$, $l_2 = kd_2$ and the characteristic impedance is Z_0, in which k is the wave number and $d_{1,2}$ is the physical lengths of the transmission lines. Furthermore, the resistance of reactance component which consists the capacitor C or the inductor L is $X = 1/\omega C$ or $X = \omega L$ between the two transmission lines. Based on the scattering matrix method, the circuit can exhibit an ideal unidirectional performance at the spontaneous PT-symmetry breaking point by tuning the transmission lines between the lumped elements. Additionally, the resistance of the reactance component can alter the bandwidth of the unidirectional invisibility flexibly.

The system in reference [31] has the exactly same structure as our dimer. An interesting result of two-port scattering in this paper is that at specific ω values, the transmittance becomes $t = 1$, while at the same time one of the reflectances vanishes. Hence, the scattering for this direction of incidence is flux conserving and the structure is unidirectionally transparent. Periodic repetition of the PT-symmetric unit will result in the creation of unidirectionally transparent frequency bands. We recommend these circuit structures for the study of unidirectional invisibility in the electronic domain. With proper optimization and design techniques, all these circuits can be implemented on IC.

8.2 Discussion on Topological PT-symmetric Electronics

Topological properties experience an intriguing degree of diversification when they are combined with PT symmetry. Therefore, there have been considerable efforts devoted to studying topological insulators under the context of PT symmetry. Here, we would like to give some discussions about studying topological effects with non-Hermitian topological electronic circuits.

Prior arts [33, 34, 35, 36] have used PT-symmetric electronic circuits to demonstrate various topological effects, such as topological defect engineering, topological insulating phase, and topological wireless power transfer. So far, these experiments have been focused on low-frequency platform, i.e., printed circuit board. A general 1-D PT-symmetric Su-Schrieffer-Heeger (SSH) tight-binding model is illustrated in Supplementary Figure 23**a**, which is based on a chain with alternating hop-

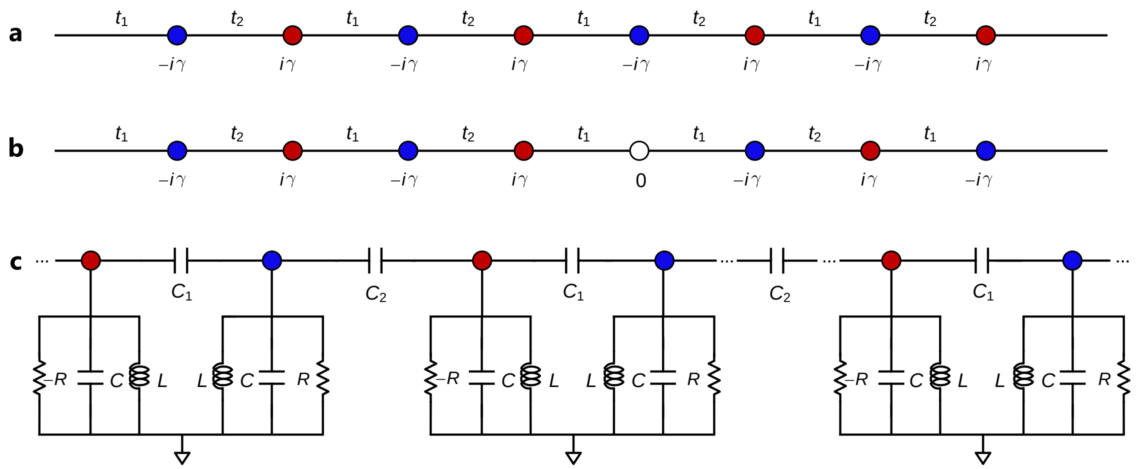

Supplementary Figure 23: Theoretical hopping model and circuit diagram for topological electronics, which is modified from ref [33]. **a**. Bulk model with two sites per unit cell (red and blue), with alternating hoppings t_1 and t_2 and on-site gain/loss terms $\pm i\gamma$. **b**. Insertion of the PT symmetric defect (white empty circle). **c**. Circuit diagram of the experimental implementation. The hoppings are realized by capacitors C_1, C_2, the on-site gain and loss by resistive elements $-R$, R. The inductor L or capacitor C tunes the resonance frequency of the circuit.

ping t_1 and t_2, and an alternating on-site gain and loss term $\pm i\gamma$. The non-Hermitian SSH model is represented by the admittance matrix, also termed circuit Laplacian $J(\omega)$ [37] of the circuit. The detailed theoretical analysis of such non-Hermitian SSH models can be found in prior works [33, 37]. Supplementary Figure 23**c** shows the corresponding circuit design of the 1-D topological chain, where the hoppings between non-Hermitian cells are represented by capacitors C_1, C_2, and the on-site gain and loss are realized by resistive elements. In order to design a manifestly PT-symmetric topological midgap state in a waveguide system, a defect site can be inserted into chain as shown in Supplementary Figure 23**b**. Realize such a chain in CMOS is conceptually straightforward. However, there are two concerns we want to discuss. First, the parasitics are universal in IC designs. Therefore, the parasitics between the connection of two adjacent non-Hermitian units should be minimized. Second, nonlinearities are also common in ICs. These non-idealities should be included into the theoretical analysis of the 1-D topological chain.

References

[1] J. Schindler, Z. Lin, J. M. Lee, H. Ramezani, F. M. Ellis, and T. Kottos, "PT-symmetric electronics," *Journal of Physics A: Mathematical and Theoretical*, vol. 45, no. 44, p. 444029, oct 2012.

[2] P.-Y. Chen, M. Sakhdari, M. Hajizadegan, Q. Cui, M. M.-C. Cheng, R. El-Ganainy, and A. Alù, "Generalized parity–time symmetry condition for enhanced sensor telemetry," *Nature Electronics*, vol. 1, no. 5, May 2018.

[3] H. Jeon and K. W. Kobayashi, "Linear voltage controlled variable resistor using body potential in soi process," *IEEE Microwave and Wireless Components Letters*, vol. 26, no. 10, pp. 816–818, 2016.

[4] R. S. SITARAM and W. G. TOWNSEND, "A voltage-controlled variable-resistance mosfet," *International Journal of Electronics*, vol. 38, no. 2, pp. 253–257, 1975. [Online]. Available: https://doi.org/10.1080/00207217508920395

[5] B. Razavi, "A 300-GHz Fundamental Oscillator in 65-nm CMOS Technology," *IEEE Journal of Solid-State Circuits*, vol. 46, no. 4, pp. 894–903, 2011.

[6] T. Djurhuus, V. Krozer, J. Vidkjaer, and T. K. Johansen, "Nonlinear analysis of a cross-coupled quadrature harmonic oscillator," *IEEE Transactions on Circuits and Systems I: Regular Papers*, vol. 52, no. 11, pp. 2276–2285, 2005.

[7] Y. D. Chong, L. Ge, and A. D. Stone, "$\mathcal{PT}$-Symmetry Breaking and Laser-Absorber Modes in Optical Scattering Systems," *Phys. Rev. Lett.*, vol. 106, p. 093902, Mar 2011.

[8] A. Mostafazadeh, "Spectral Singularities of Complex Scattering Potentials and Infinite Reflection and Transmission Coefficients at Real Energies," *Phys. Rev. Lett.*, vol. 102, p. 220402, Jun 2009.

[9] W. Chen, C. Wang, B. Peng, and L. Yang, "Non-hermitian physics and exceptional points in high-quality optical microresonators," in *Ultra-high-q Optical Microcavities*. World Scientific, 2021, pp. 269–313.

[10] C. Wang, Z. Fu, and L. Yang, "Non-hermitian physics and engineering in silicon photonics," in *Silicon Photonics IV*. Springer, 2021, pp. 323–364.

[11] W. Cao, X. He, A. Chakrabarti, and X. Zhang, "NeuADC: Neural Network-Inspired RRAM-Based Synthesizable Analog-to-Digital Conversion with Reconfigurable Quantization Support," in *2019 Design, Automation Test in Europe Conference Exhibition (DATE)*, 2019, pp. 1477–1482.

[12] W. Cao, X. He, A. Chakrabarti, and X. Zhang, "NeuADC: Neural Network-Inspired Synthesizable Analog-to-Digital Conversion," *IEEE Transactions on Computer-Aided Design of Integrated Circuits and Systems (TCAD)*, vol. 39, no. 9, pp. 1841–1854, 2020.

[13] W. Cao, L. Ke, A. Chakrabarti, and X. Zhang, "Neural Network-Inspired Analog-to-Digital Conversion to Achieve Super-Resolution with Low-Precision RRAM Devices," in *2019 IEEE/ACM International Conference on Computer-Aided Design (ICCAD)*, 2019, pp. 1–7.

[14] W. Cao, L. Ke, A. Chakrabarti, and X. Zhang, "Evaluating Neural Network-Inspired Analog-to-Digital Conversion With Low-Precision RRAM," *IEEE Transactions on Computer-Aided Design of Integrated Circuits and Systems (TCAD)*, vol. 40, no. 5, pp. 808–821, 2021.

[15] W. Cao, Y. Zhao, A. Boloor, Y. Han, X. Zhang, and L. Jiang, "Neural-pim: Efficient processing-in-memory with neural approximation of peripherals," *IEEE Transactions on Computers*, pp. 1–1, 2021.

[16] F. Lv, X. Zheng, F. Zhao, J. Wang, S. Yue, Ziqiang Wang, W. Cao, Y. He, C. Zhang, H. Jiang, and Z. Wang, "A power scalable 2–10 gb/s pi-based clock data recovery for multilane applications," *Microelectronics Journal*, vol. 82, pp. 36–45, 2018.

[17] B. Razavi, "A study of phase noise in cmos oscillators," *IEEE Journal of Solid-State Circuits*, vol. 31, no. 3, pp. 331–343, 1996.

[18] H.-C. Chang, X. Cao, U. Mishra, and R. York, "Phase noise in coupled oscillators: theory and experiment," *IEEE Transactions on Microwave Theory and Techniques*, vol. 45, no. 5, pp. 604–615, 1997.

[19] G. Li, L. Liu, Y. Tang, and E. Afshari, "A low-phase-noise wide-tuning-range oscillator based on resonant mode switching," *IEEE Journal of Solid-State Circuits*, vol. 47, no. 6, pp. 1295–1308, 2012.

[20] S. A.-R. Ahmadi-Mehr, M. Tohidian, and R. B. Staszewski, "Analysis and design of a multi-core oscillator for ultra-low phase noise," *IEEE Transactions on Circuits and Systems I: Regular Papers*, vol. 63, no. 4, pp. 529–539, 2016.

[21] A. ElSayed and M. Elmary, "Low-phase-noise lc quadrature vco using coupled tank resonators in a ring structure," *IEEE Journal of Solid-State Circuits*, vol. 36, no. 4, pp. 701–705, 2001.

[22] Schindler and J. Caulfield, "PT-Symmetric Electronics," *Masters Theses, https://wesscholar.wesleyan.edu/etd_mas_theses/42*, vol. 42, 2013.

[23] A. Mirzaei, M. E. Heidari, R. Bagheri, S. Chehrazi, and A. A. Abidi, "The Quadrature LC Oscillator: A Complete Portrait Based on Injection Locking," *IEEE Journal of Solid-State Circuits*, vol. 42, no. 9, pp. 1916–1932, 2007.

[24] G. Cusmai, M. Repossi, G. Albasini, A. Mazzanti, and F. Svelto, "A Magnetically Tuned Quadrature Oscillator," *IEEE Journal of Solid-State Circuits*, vol. 42, no. 12, pp. 2870–2877, 2007.

[25] J. Kim, J. Kim, G. Kim, and D. Jeong, "A Fully Integrated 0.13- μm CMOS 40-Gb/s Serial Link Transceiver," *IEEE Journal of Solid-State Circuits*, vol. 44, no. 5, pp. 1510–1521, 2009.

[26] D. Guermandi, P. Tortori, E. Franchi, and A. Gnudi, "A 0.83-2.5-GHz continuously tunable quadrature VCO," *IEEE Journal of Solid-State Circuits*, vol. 40, no. 12, pp. 2620–2627, 2005.

[27] N. Bender, S. Factor, J. D. Bodyfelt, H. Ramezani, D. N. Christodoulides, F. M. Ellis, and T. Kottos, "Observation of Asymmetric Transport in Structures with Active Nonlinearities," *Phys. Rev. Lett.*, vol. 110, p. 234101, Jun 2013.

[28] D. L. Sounas, J. Soric, and A. Alù, "Broadband passive isolators based on coupled nonlinear resonances," *Nature Electronics*, vol. 1, pp. 113–119, 2018.

[29] L. Shao, W. Mao, S. Maity, N. Sinclair, Y. Hu, L. Yang, and M. Lončar, "Non-reciprocal transmission of microwave acoustic waves in nonlinear parity–time symmetric resonators," *Nature Electronics*, vol. 3, no. 5, pp. 267–272, May 2020.

[30] B. Lv, J. Fu, B. Wu, R. Li, Q. Zeng, X. Yin, Q. Wu, L. Gao, W. Chen, Z. Wang, Z. Liang, A. Li, and R. Ma, "Unidirectional invisibility induced by parity-time symmetric circuit," *Scientific Reports*, vol. 7, no. 1, p. 40575, Jan 2017.

[31] Z. Lin, J. Schindler, F. M. Ellis, and T. Kottos, "Experimental observation of the dual behavior of $\mathcal{PT}$-symmetric scattering," *Phys. Rev. A*, vol. 85, p. 050101, May 2012.

[32] F. R. Humire and E. Lazo, "$\mathcal{PT}$-symmetric direct electrical transmission lines: Localization behavior," *Phys. Rev. E*, vol. 100, p. 022221, Aug 2019.

[33] A. Stegmaier, S. Imhof, T. Helbig, T. Hofmann, C. H. Lee, M. Kremer, A. Fritzsche, T. Feichtner, S. Klembt, S. Höfling, I. Boettcher, I. C. Fulga, O. G. Schmidt, M. Greiter, T. Kiessling, A. Szameit, and R. Thomale, "Topological defect engineering and PT-symmetry in non-hermitian electrical circuits," *Preprint at*, p. https://arxiv.org/abs/2011.14836, 2020.

[34] H. Zhao, X. Qiao, T. Wu, B. Midya, S. Longhi, and L. Feng, "Non-hermitian topological light steering," *Science*, vol. 365, no. 6458, pp. 1163–1166, 2019.

[35] S. Liu, S. Ma, C. Yang, L. Zhang, W. Gao, Y. J. Xiang, T. J. Cui, and S. Zhang, "Gain- and Loss-Induced Topological Insulating Phase in a Non-Hermitian Electrical Circuit," *Phys. Rev. Applied*, vol. 13, p. 014047, Jan 2020.

[36] L. Zhang, Y. Yang, Z. Jiang, Q. Chen, Q. Yan, Z. Wu, B. Zhang, J. Huangfu, and H. Chen, "Topological wireless power transfer," *Preprint at*, p. https://arxiv.org/abs/2008.02592, 2020.

[37] C. H. Lee, S. Imhof, C. Berger, F. Bayer, J. Brehm, L. W. Molenkamp, T. Kiessling, and R. Thomale, "Topolectrical circuits," *Communications Physics*, vol. 1, no. 1, p. 39, Jul 2018.
